# Circadian clock crosstalks with autism

**DOI:** 10.1002/brb3.3273

**Published:** 2023-10-08

**Authors:** Ekin Yurdakul, Yaman Barlas, Kutlu O. Ulgen

**Affiliations:** ^1^ Department of Chemical Engineering Bogazici University, Biosystems Engineering Laboratory Istanbul Turkey; ^2^ Department of Industrial Engineering Bogazici University, Socio‐Economic System Dynamics Research Group (SESDYN) Istanbul Turkey

**Keywords:** ASD, bioinformatics, circadian rhythmicity, neurodevelopmental diseases, protein–protein interaction network

## Abstract

**Background:**

The mechanism underlying autism spectrum disorder (ASD) remains incompletely understood, but researchers have identified over a thousand genes involved in complex interactions within the brain, nervous, and immune systems, particularly during the mechanism of brain development. Various contributory environmental effects including circadian rhythm have also been studied in ASD. Thus, capturing the global picture of the ASD‐clock network in combined form is critical.

**Methods:**

We reconstructed the protein–protein interaction network of ASD and circadian rhythm to understand the connection between autism and the circadian clock. A graph theoretical study is undertaken to evaluate whether the network attributes are biologically realistic. The gene ontology enrichment analyses provide information about the most important biological processes.

**Results:**

This study takes a fresh look at metabolic mechanisms and the identification of potential key proteins/pathways (ribosome biogenesis, oxidative stress, insulin/IGF pathway, Wnt pathway, and mTOR pathway), as well as the effects of specific conditions (such as maternal stress or disruption of circadian rhythm) on the development of ASD due to environmental factors.

**Conclusion:**

Understanding the relationship between circadian rhythm and ASD provides insight into the involvement of these essential pathways in the pathogenesis/etiology of ASD, as well as potential early intervention options and chronotherapeutic strategies for treating or preventing the neurodevelopmental disorder.

## Graphical abstract

This study highlights the relationship between autism and the circadian clock using a five‐step approach: 1. Collection of Protein‐Protein Interaction (PPI) Data, 2. Reconstruction of autism, circadian and combined circadian‐autism Protein‐Protein Interaction Networks (PPINs), 3. Conducting topological analysis and Gene Ontology enrichment analysis, 4. Conducting network decompositon analysis, and 5. Analyzing the results regarding the connection between autism and the circadian clock, providing insights for future chronotherapeutic strategies.

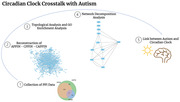

## 1 INTRODUCTION

The suprachiasmatic nucleus (SCN), located in the anterior hypothalamus, regulates the mammalian circadian clock, and both cortisol and melatonin periods during the day (Buijs et al., 2003). The secretion of the melatonin hormone increases following the perception of darkness by the SCN (Buijs et al., 2003). The perception of light during sleep patterns may lead to the suppression of melatonin hormone and the disruption of the circadian rhythm. For individuals who are accustomed to artificial light at night during night shifts, the circadian rhythm may become disrupted (Dickerman & Liu, 2012). Exposure to artificial light at night has been associated with oxidative stress and DNA damage (Dickerman & Liu, 2012). In a clinical research study (Martinez‐Cayuelas et al., 2022), individuals with autism spectrum disorder (ASD) were found to exhibit distinct melatonin secretion patterns, which could potentially influence sleep challenges and circadian rhythm disruptions. In another clinical research (Ballester‐Navarro et al., 2021), it has been stated that both normal and abnormal sleep‐wake rhythms in individuals ranging in age from 18 to 41 years (autistic cases) might be associated with specific gene variants in the circadian clock (PER1) and melatonin pathway (*N*‐acetyl serotonin *O*‐methyltransferase [ASMT]) systems. A growing body of evidence indicates that the misalignment of the circadian system is associated with various chronic diseases including cardiovascular, metabolic, and neuropsychiatric disorders such as schizophrenia and ASD (Lorsung et al., 2021).

The neuropsychiatric disorder ASD is primarily caused by genetic factors (e.g., mutations in genes, chromosomal abnormalities, and copy number variations [CNVs]) and environmental factors as studied extensively. Several research studies have demonstrated a direct correlation between ASD and circadian rhythm disorders (Jin et al., 2018) (Abdul et al., 2022). Pregnant women who work night shifts may experience stress and disrupted circadian rhythms, and potentially leading to altered hormone levels, including melatonin and cortisol. These altered hormone levels are considered risk factors for ASD (Jin et al., 2018) (Taylor & Corbett, 2014), as indicated by studies such as those conducted by Nir et al. (1995) and Corbett et al. (2009). Environmental conditions including shift work and sleep disorders can impact maternal circadian rhythms (Wong et al., 2022), potentially affecting the development of fetal circadian rhythmicity (Sorensen et al., 2020). Preterm birth is closely associated with ASD (Fitzgerald et al., 2020) (Allotey et al., 2018). Besides, preterm babies experience constant exposure to bright light and loud noises in neonatal intensive care units, which can disrupt their sleep patterns and impact their circadian development (Sorensen et al., 2020). The ultimate effect is the dysregulation of hormone levels including melatonin and glucocorticoids; this dysregulation has an impact on growth, development, and other health outcomes such as the increased risk of autism and impaired learning, vision, and neurobehavioral abnormalities (Allotey et al., 2018; Sorensen et al., 2020). ASD and attention deficit hyperactivity disorder have also been associated with changes in the functioning of various neurotransmitters like gamma‐aminobutyric acid (GABA), norepinephrine, dopamine, and serotonin (Marotta et al., 2020; Eissa et al., 2018). These studies point out a causative link among the HPA axis, circadian rhythm disruption, and ASD.

For several behavioral complications of ASD patients, sleep and circadian rhythm disturbances have been given as explanatory factors (Yavuz‐Kodat et al., 2020). Melatonin synthesis, essential for neurodevelopment, has been found to be low in autistic children (Kulman et al., 2000). Mutations in the ASMT enzyme of the melatonin synthesis pathway are reported in ASD patients pointing to a genetic link (Jonsson et al., 2010). Melatonin synthesis begins with tryptophan, followed by intermediates of 5‐hydroxytryptophan, serotonin, and *N*‐acetyl serotonin, respectively (Barrenetxe et al., 2004). Differences are seen in serotonin levels in the blood of children with autism. The blood levels of serotonin in children with advanced autism are higher than those of children with less advanced autism (Abdulamir et al., 2018). The disruptions in melatonin secretion and the serotonin system involved in the melatonin pathway are remarkable in ASDs, pointing out its association with circadian rhythm. The rhythmicity in the secretion of neurohormones, including cortisol, prolactin, and thyroid‐stimulating hormone, also has a role in the pathology of autism (Nir et al., 1995; Taylor & Corbett, 2014). A dysfunctional HPA axis and aberrant cortisol levels are observed in autistic children (Lorsung et al., 2021). Mean cortisol levels are found to be higher among ASD patients with IQ < 55 (extremely low functioning ASD individuals), compared to those with IQ ≥ 70 (Putnam, 2015; Kidd et al., 2012). Activity‐dependent changes in the efficacy of synaptic communication become particularly important in circadian rhythm disorders, which can increase the susceptibility to ASD during early neurodevelopment (Yenen & Çak, 2020) (Ebert & Greenberg, 2013).

This study focuses on the reconstruction of protein–protein interaction (PPI) networks of ASD and circadian rhythm to understand the connection between autism and the circadian clock. Up to this time, most published research has concentrated on the behavioral characteristics of people with ASD. Experimentally, animal models have been employed to explore the complex history of ASD, as it was impossible to create human neural cell cultures that could proliferate indefinitely (Pensado‐López et al., 2020). Hence, it is of utmost significance to capture the comprehensive overview of these two networks in the combined form to understand the crosstalk between ASD and circadian rhythmicity as well as other metabolic pathways. A graph theoretical analysis is performed to determine whether the network properties are biologically feasible. The gene ontology (GO) enrichment analysis provides detailed information about the essential cellular processes. This study presents a comprehensive circadian‐ASD network indicating key components, that facilitate a deeper understanding of shared mechanisms underlying circadian rhythm disorder‐related pathophysiology of ASD.

## 2 MATERIALS AND METHODS

### 2.1 *Homo sapiens* protein–protein introduction data

*Homo sapiens* PPI data are obtained from the BioGRID database (Stark et al., 2006). Within these data, only experimental system types, specifically physical interactions, are used, and genetic interactions are excluded. As the confidence score is connected to the reliability of PPI from physical data, interactions with confidence scores below 0.85 are removed. In cases where a different scoring system produces data with scores exceeding 1, the average of interaction scores within the same publication is calculated. Interaction data with scores higher than 1.5 times the calculated average are then selected. As a result, a set of 102,991 PPI data is collected as reliable for further analysis. A Python code is developed to facilitate filtering process. Additionally, a dataset of physical PPI (experimental) with confidence scores higher than 0.85, obtained from the String database, is appended (106,669 PPIs). The total number of unique PPIs is 209,658.

### 2.2 Construction of circadian PPI networks

The “circadian” term is used to search the Gene Ontology Consortium web page (Carbon et al., 2009) to create a dataset comprising GO‐terms related to the circadian function. All three types of GO terms (biological process, molecular function, and cellular component) are thoroughly examined to identify the terms that demonstrate significant associations with each studied protein set. A total of 7253 GO‐terms are linked to the “Circadian” term for *H. sapiens*, among these, there are 34,926 proteins associated with molecular function, 38,012 proteins associated with the cellular component, and 35,556 proteins associated with biological process. 26,504 proteins are common across all three types of GO terms under the “circadian” term. The PPIs of these shared 26,504 proteins are determined within the interacting protein set, resulting in 77,594 PPIs. Using these pairwise proteins, a PPI network is reconstructed, referred to as “circadian PPI network 1 (CPPIN1).” Subclusters within this network are identified using Cytoscape software for further analysis.

Moreover, after careful literature research, an additional refined dataset consisting of 1435 human circadian gene products is identified (Li et al., 2016) and processed as explained above, resulting in 1417 PPIs and referred to as “CPPIN2.”

### 2.3 Construction of autism PPI networks

Through a literature review, a total of 106 susceptibility genes associated with autism in *H. sapiens* are identified (Iakoucheva et al., 2019). Additionally, a list of 3590 GO ID numbers linked to these susceptibility genes is compiled. The network created from 106 gene products is referred to as “Autism PPI Network 1 (APPIN1).” The first neighbors (1444 proteins) are found using the collection of unique PPI data (comprising 27,008 interactions). Next, the complete set of autism‐related proteins (1550) is searched within the interacting protein set, discovering 31,383 PPIs. These pairwise proteins are used to reconstruct a PPI network named “APPIN2.”

Next, an additional autism dataset (comprising 191 autism risk factors) is examined (Corominas et al., 2014). This dataset is then combined with the existing 106 proteins in APPIN1, resulting in the reconstruction of a third network, named “APPIN3.” This dataset contains 287 unique proteins connected by 193 PPIs. Furthermore, other data from Corominas et al. (2014), having 291 autism‐related risk proteins, are selected and then combined with 106 proteins of Iakoucheva et al. (2019), to construct APPIN4, where 397 unique proteins resulted in 263 PPIs. Finally, these three datasets (APPIN4 + APPIN3 + APPIN1) are combined to reconstruct APPIN5 that contains 484 PPIs among a total of 559 unique proteins.

### 2.4 Construction of circadian and autism PPI joint network

In the autism and circadian joint network, the proteins from APPIN5 and CPPIN2 are combined, resulting in the construction of a network consisting of 1844 proteins and 2394 interactions. The more comprehensive APPIN5 is selected, encompassing circadian proteins within its clusters. CPPIN2 is chosen due to its more specific circadian network compared to CPPIN1. This combined network is named combined circadian–autism PPI (CAPPIN) and has undergone a detailed study.

### 2.5 Topological analysis and clusters and GO enrichment analysis

Graph theoretical analysis is used to conduct topological analysis of the reconstructed PPI networks. Properties such as node degree, hubs (highly connected nodes), and shortest path lengths among indirectly connected nodes, network diameter, and mean path length are determined using the Network Analyzer plugin (ver. 2.7 and 4.4.6) (Doncheva et al., 2012) within Cytoscape (ver. 3.6.1 and 3.9.0) (Shannon et al., 2003). The list of binary interacting proteins is given as the input, and the collected output includes different topological network parameters such as network clustering coefficient, network diameter, network radius, characteristic path length, average number of neighbors, network density, and node degree.

MCODE plug‐in (v1.5.1 and v2.0.0) is utilized to identify clusters (highly interconnected regions) within the network (Bader & Hogue, 2003). The node score cutoff is set to 0.2 for cluster expansion, the *K*‐core value is set to 2, and the maximum depth from seed is set to 100. The GO Tool developed by Princeton University (Boyle et al., 2004) is used to analyze the GO terms through enrichment analysis. The significance level is set to 0.05, indicating that only terms enriched with a *p*‐value of .05 or lower are taken into consideration.

### 2.6 Network decomposition analysis (NDA)

To comprehend the information flow (topology) within the circadian‐autism PPIN, linear paths connecting the center protein involved in circadian rhythm to the transcription factor in the nucleus related to ASD are determined using the NetSearch algorithm (Steffen et al., 2002). NetSearch draws all possible linear paths of a specified length through the interaction map, starting at any membrane protein and ending at any DNA‐binding protein. Hence, the protein–protein interaction graph (comprising directed interactions) is used as the input for the NetSearch algorithm. Additionally, the other inputs (the circadian rhythm‐related center protein) and the output (an autism‐related transcription factor representing a cellular response) need also be defined for the linear path calculations. This linear path analysis is conducted to obtain insights into network crosstalk, and the molecules' involvement in the CAPPIN pathways.

## 3 RESULTS

### 3.1 Reconstruction and topological analysis of PPI networks of circadian and autism systems

**FIGURE 1:**
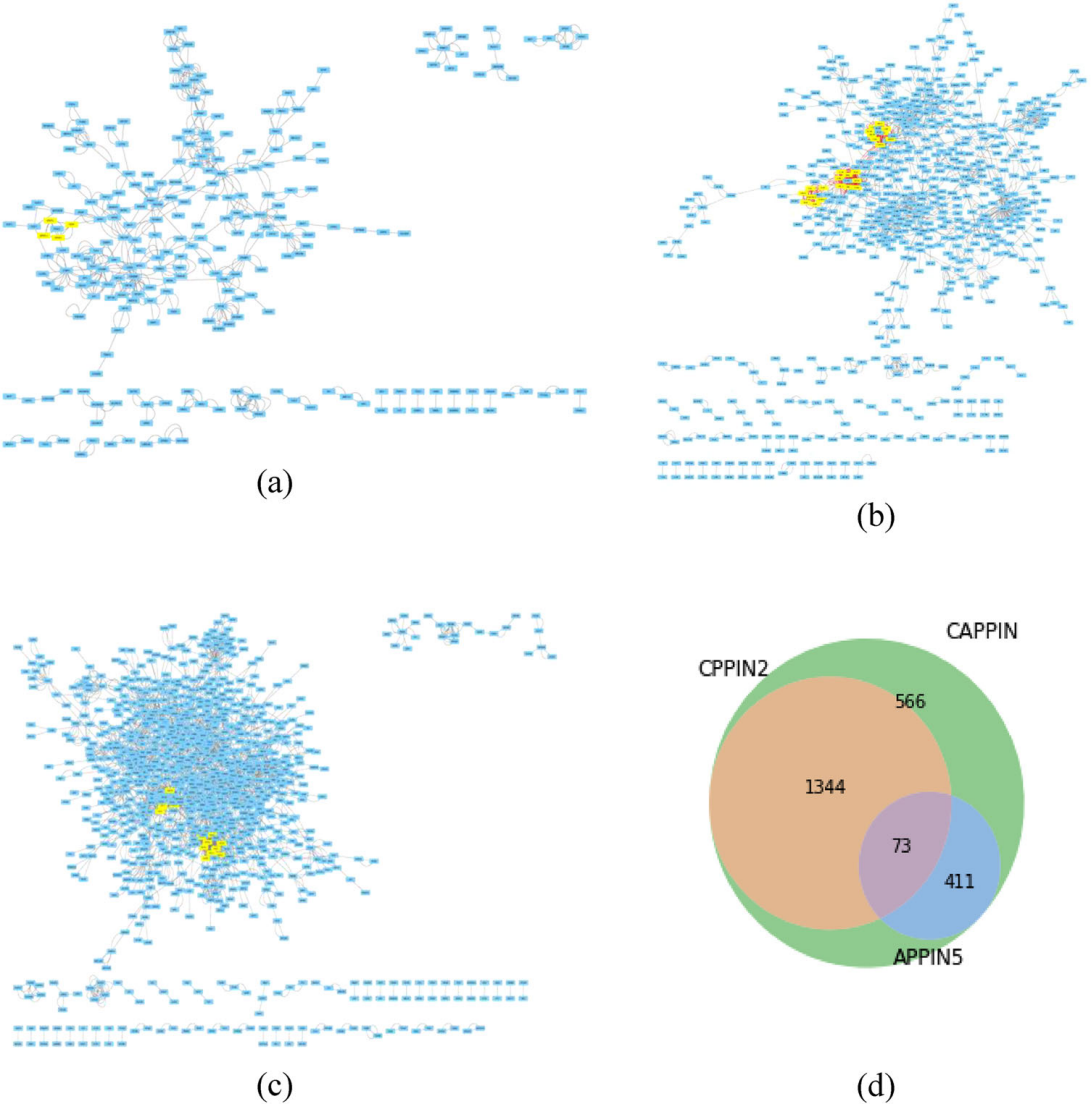
Protein–protein interaction networks and Venn diagram: (a) APPIN5: This significant network focuses on interactions and highlights hub proteins and their connections related to autism spectrum disorder (ASD); (b) CPPIN2: This network emphasizes circadian clock‐related interactions; (c) combined circadian–autism protein–protein interaction network (CAPPIN): The CAPPIN visualizes comprehensive protein–protein interactions, combining APPIN5 and CPPIN2, showcasing the significant interactions between autism and the circadian clock; (d) Venn diagram of interactions: This diagram provides an overview of the overlaps and unique interaction numbers between these protein interaction networks.

The circadian (CPPIN), autism (APPIN), and CAPPIN networks are created by following the steps outlined in Section 2 (Figure 1). A graph theoretical analysis is conducted to calculate various topological properties, including the clustering coefficient, node connectivity, the shortest path lengths among indirectly connected nodes, network diameter, centrality measures, and characteristic path length. The clustering coefficient indicates the degree to which a network exhibits a clustering tendency (Masuda et al., 2018). The clustering coefficients for CPPIN2, APPIN5, and CAPPIN are 0.139, 0.225, and 0.151, respectively (as presented in Tables 1 and 2), and these values are consistent with the literature‐reviewed values of normal human cells. The network clustering coefficients for bone cells are estimated as 0.217 and 0.261 for normal and cancer PPIN, respectively (Rahman et al., 2013; Sahoo et al., 2016). In another study from the literature (Dahiya et al., 2019), the clustering coefficient of the human brain‐specific PPIN is 0.108. The diameter of the network represents the distance between the two farthest points (Junker & Schreiber, 2008). Compared to the same dataset of bone cells, where the bone PPIN diameter is 7 (Rahman et al., 2013; Sahoo et al., 2016), the diameter of circadian, autism, and combined PPIN diameters are slightly higher, that is, 18, 18, and 14, respectively. Closeness centrality indicates the distance of a protein in the network to all other proteins (Junker & Schreiber, 2008). The change in the closeness centrality with the number of neighboring proteins demonstrates a linear relationship. When comparing these values with the literature, the characteristic path length and network diameter closely resemble those reported for various human‐related networks (Table 3). Based on these network properties, the CPPINs, APPINs, and CAPPIN are considered small‐world networks, similar to many other complex biological networks (Latora & Marchiori, 2001; Watts & Strogatz, 1998).

**TABLE 1:** The result of analyzing autism protein–protein interaction (PPI) networks.

	APPIN1	APPIN2	APPIN3	APPIN4	APPIN5 (APPIN4 + APPIN3 + APPIN1)
Number of proteins	106	1550	287	397	559
P–P interactions	44	27008	193	263	484
Clustering coefficient	0.115	0.357	0.170	0.175	0.225
Number of nodes	31	1537	101	165	259
Network diameter	6	11	13	15	18
Characteristic path length	2.598	3.583	4.279	6.138	6.745
Network density	0.054	0.013	0.021	0.014	0.015
Network heterogeneity	0.699	1.166	0.779	0.692	0.691

**TABLE 2:** The result of analyzing circadian and circadian–autism combined protein–protein interaction (PPI) networks.

	CPPIN1	CPPIN2	New CAPPIN (APPIN5 + CPPIN2)
Number of proteins	26,504	1435	1844
P–P interactions	77,594	1417	2394
Clustering coefficient	0.123	0.139	0.151
Number of nodes	9333	657	980
Network diameter	13	18	14
Characteristic path length	4.611	6.731	5.896
Network density	0.001	0.005	0.004
Network heterogeneity	1.671	1.026	0.954

Abbreviation: CAPPIN, combined circadian–autism protein–protein interaction network.

**TABLE 3:** Graph theory results of *Homo sapiens* protein–protein interaction (PPI) networks in literature.

Model PPI network	Insulin	Wnt/PCP	Random	Hypertension	DIP
Clustering coefficient	–	–	0.16	–	0.2056
Number of nodes	498	1952	8448	535	1065
Network diameter	5	13	13	12	21
Characteristic path length	2.9	4.61	4.39	5.23	6.80
Reference	Durmuş Tekir et al. (2010)	Nalbantoglu et al. (2011)	Xu et al. (2011)	Ran et al. (2013)	Wu et al. (2005)

The properties of the nine main circadian genes (PER1, PER2, PER3, CLOCK, ARNTL [BMAL1], CRY1, CRY2, CSNK1E, and NPAS2) in CPPIN2 are tabulated in Table 4. Closeness centrality demonstrates the distance of a protein to other proteins (Junker & Schreiber, 2008) which indicates the effective spread of information through the network. Among these main nine circadian proteins, CSNK1E stands out with the highest closeness centrality value (0.219) signifying its role in efficiently facilitating the flow of information between the proteins. In PPI networks, radiality is associated with the probability of a protein being functionally connected to other proteins. Therefore, the CSNK1E, with the highest radiality value among its counterparts, emerges as the functionally relevant entity in relation to other primary circadian proteins.

**TABLE 4:** Main nine circadian genes properties in the protein–protein interaction (PPI) circadian network.

Gene name	CSNK1E	CRY1	CRY2	ARNTL	PER1	CLOCK	NPAS2	PER2	PER3
Average shortest path length	4.564	5.511	5.511	5.512	5.514	5.514	5.514	5.514	6.507
Clustering coefficient	0.275	0.750	0.750	0.929	0.952	0.952	0.952	0.952	1.000
Closeness centrality	0.219	0.181	0.181	0.181	0.181	0.181	0.181	0.181	0.154
Degree	34	21	19	18	18	15	15	15	4
Neighborhood connectivity	8.563	8.444	8.444	9.375	9.857	9.857	9.857	9.857	9.000
Radiality	0.802	0.749	0.749	0.749	0.749	0.749	0.749	0.749	0.694

### 3.2 Network's hub protein analysis

The node connectivity identifies highly connected proteins that participate in significant numbers of interactions and hold critical roles in the organization of the cellular protein interaction network. The top 10 highly connected proteins, referred to as hub proteins, are listed in Table 5A and B.

**TABLE 5a:** The highest degree proteins in the autism protein–protein interaction (PPI) networks.

APPIN1		APPIN2		APPIN3		APPIN4		APPIN5	
HubProt	Dg.	HubProt	Dg.	HubProt	Dg.	HubProt	Dg.	HubProt	Dg.
CREBBP	11	RPS27A	496	CTNNB1	18	CREBBP	22	CREBBP	24
CTNNB1	8	UBB	302	DLG2	16	CTNNB1	13	CTNNB1	21
ANK2	6	RPS3	297	NRXN1	14	CTBP1	13	CTBP1	17
SIN3A	6	UBA52	296	CREBBP	14	DYNC1H1	12	DLG2	16
TBL1XR1	5	RPS2	292	HRAS	10	SEPT6	12	EIF4E	16
TCF7L2	4	RPS8	290	SHANK2	10	TCF4	11	NRXN1	16
PHF12	4	RPS6	286	QPRT	9	PSME3	10	STX1A	15
PHF21A	4	UBC	279	STX1A	8	STX1A	10	DYNC1H1	14
TCF4	4	RPS16	279	APC	8	SNAP29	10	SNAP29	13
DYNC1H1	4	RPSA	274	ANK2	8	SIN3A	10	HRAS	12

**TABLE 5b:** The highest degree proteins in circadian and combined circadian–autism protein–protein interaction (PPI) networks.

CPPIN1		CPPIN2		CAPPIN	
HubProt	Degree	HubProt	Degree	HubProt	Degree
TRIM28	656	UBE2B	41	UBE2B	56
RPS27A	625	UBE2C	41	UBE2C	56
UBB	456	RPL14	41	RPL14	47
GNB4	424	RPS5	38	RPS23	41
GNB3	422	RPS23	37	RPS5	41
GNB5	421	CSNK1E	34	GNG2	40
UBC	404	RPS25	33	CSNK1E	37
RPS16	373	CSNK1D	29	RPS25	36
GNGT1	352	RPL3	28	CREBBP	35
RPS12	348	RPL21	28	CTNNB1	34

Abbreviation: CAPPIN, combined circadian–autism protein–protein interaction network.

In circadian network CPPN1, a crucial hub protein is the ubiquitin C or polyubiquitin‐C protein, known for its stress‐protective function (Aken et al., 2016; Bianchi et al., 2018). Circadian rhythm translation–transcription feedback loops are mediated by ubiquitination, and ubiquitination intermediates play a role in maintaining the stability of the primary circadian proteins within the circadian network (Stojkovic et al., 2014). The ribosomal proteins are highly connected entities within CPPIN2, indicating the need for a deeper exploration of the relationship between the circadian and ribosomal proteins. Similar to UBE2A, the hub enzymes UBE2B and UBE2C are involved in the pathway of protein ubiquitination, specifically as ubiquitin‐conjugating enzymes E2A, E2B, and E2C. Mutations in the UBE2A gene are known to cause X‐linked intellectual disability (ID) (De Oliveira et al., 2019), suggesting a potential link to ASD. The central component of the circadian clock, CSNK1E (Casein Kinase 1, Epsilon), determines the length of thcircadian period. It holds a high degree (34 and 37) within the circadian CPPIN and the combined network CAPPIN, respectively, and is the most interacting protein among the nine primary circadian proteins. Notably, CSNK1E is determined as a gene significantly enriched in damaging de novo mutations in ASD cases (De Rubeis et al., 2014; Takata et al., 2018).

CTNNB1 protein and CREB‐binding protein (CREBBP) exist in all autism networks, except for the APPIN2. DYNC1H1 and STX1A proteins are found in three of the five networks and ANK2, SIN3A, DLG2, NRGXN1, HRAS, SNAP29, CTBP1, and TCF4 are found in two of the five autism networks. Changes in the synapse function and gene regulation may contribute to the development of ASD. ANK2 is a high‐risk ASD gene product, and ANK2 mutations are often associated with ASD (average IQ) (Yang et al., 2019). The ANK2 gene is involved in cell motility, activation, and proliferation processes (Crawley et al., 2016; Iwakawa et al., 2015). Syntaxin‐1A (STX1A), a specific protein of the nervous system, plays a role in the docking of synaptic vesicles and the presynaptic membrane; it affects serotonin (5‐HT) release in the brain and GABAergic neurotransmission, possibly contributing to attention‐deficit/hyperactivity disorder (Wang et al., 2019). The common ASD‐related proteins SIN3A, CTNNB1, and CTBP1 interact with other shared proteins: MECP2 (associated with SIN3A), CHD8‐PTK7‐TCF7L2 (associated with CTNNB1), and EHMT1‐FOXP1 (associated with CTBP1) (Crawley et al., 2016). Different publications have indicated that mutations in the CTNNB1 gene are associated with both cancer and autism (Dong et al., 2016). The point that draws attention from these highly connected proteins is that ribosomal proteins form most of the hub proteins in the APPIN networks. The CREBBP protein takes part in the transcriptional coactivation of numerous different transcription factors. Rubinstein–Taybi syndrome (RTS), classified within the ASD family, arises from a mutation in the gene coding for CREBBP (Stef et al., 2007)). Similar to ASD, the primary symptoms of Rubinstein–Taybi disorder encompass the gradual development of cognitive and motor skills (Galéra et al., 2009). The hub protein CREBBP (Table 5A) also featured in several clusters (associated with the transcription by RNA polymerase II processes) of GO enrichment analysis of 49 common proteins of CPPIN2 and APPIN5. The relationship between autism and ribosomal genes/proteins requires further investigation. Another protein listed in Table 5A, DYNC1H1, is linked to the regulation of cellular processes. Mutations in this DYNC1H1 gene may potentially contribute to neurological disorders (Hoang et al., 2017), resulting in neuromuscular and sensory deficits (Schlager et al., 2014). TCF7L2 is linked to the Wnt‐signaling pathway and is associated with schizophrenia (Alkelai et al., 2012). With a prominent expression in the brain, TCF4 actively participates in neurodevelopment. It collaborates with class II bHLH transcription factors Math1, HASH1, and neuroD2 (Navarrete et al., 2013). The TCF4 gene is also associated with memory function and plays a regulatory role in neurodevelopmental pathways (Forrest et al., 2018). Haploinsufficiency in TBL1XR1 leads to ID with dysmorphism, though it does not consistently result in autistic behavior (Pons et al., 2015). TBL1XR1 contributes to the regulation of the Wnt/β‐catenin signaling pathway, influencing various stages of brain development and processes linked to cell fate determination and stem cell renewal (Noelanders & Vleminckx, 2017). APC protein interacts with beta‐catenin and plays a critical role in multiple cellular processes. The deletion of the APC gene was observed in individuals with autism (Barber et al., 1994). NRXN1, associated with neurodevelopmental disorders, also plays a role in ASD due to CNVs in NRXN1 gene (Kim et al., 2008).

### 3.3 Module detection and analysis of PPI networks

**FIGURE 2:**
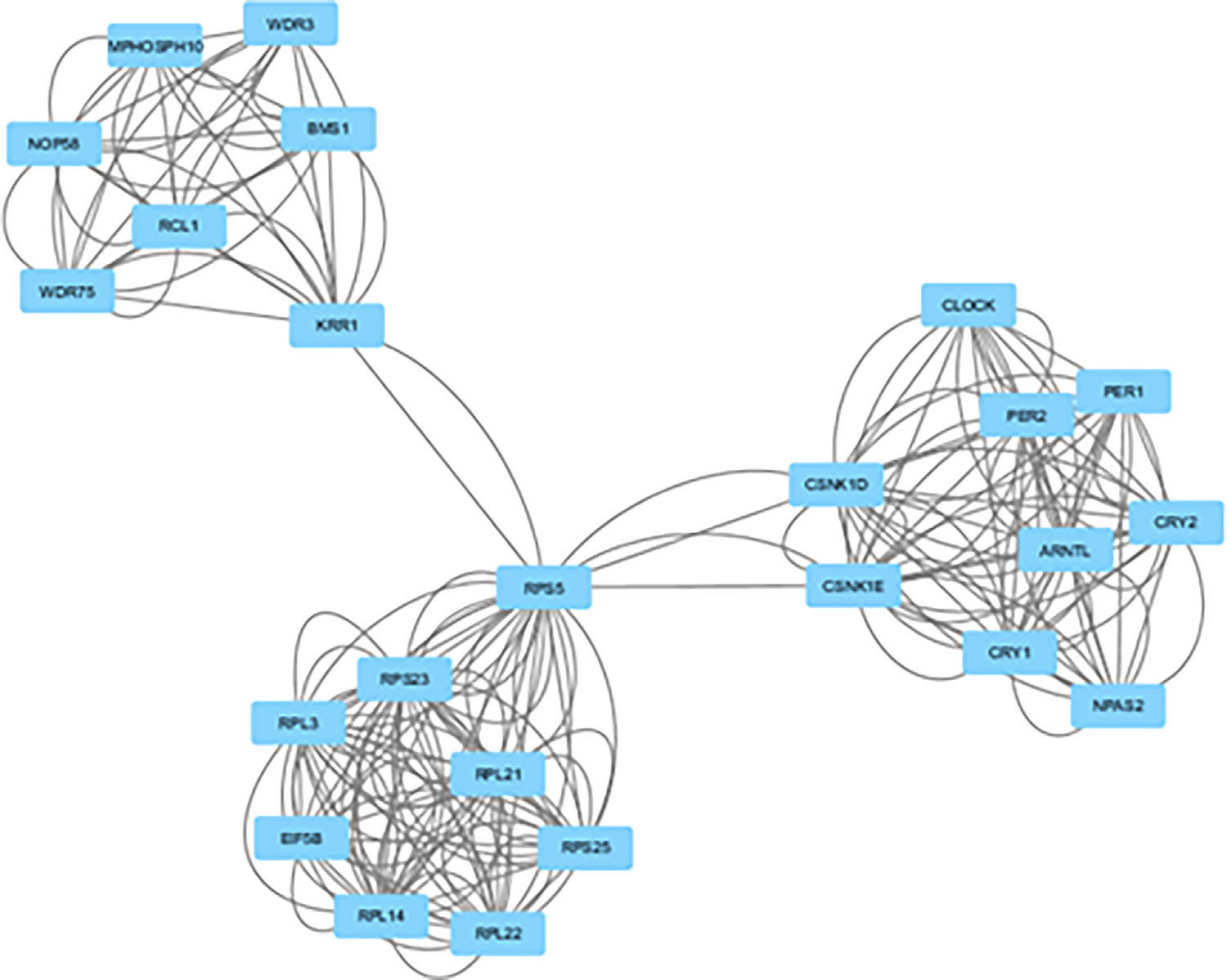
The interactome network CPPIN2 is subdivided into clusters of proteins with extensive and robust interactions. Cluster 1 from CPPIN2, which has been extracted from Cytoscape, containing essential circadian genes.

In biological systems, scale‐free networks are composed of clustered regions called modules (Bader & Hogue, 2003). CPPIN2 is divided into modules using the “Cytoscape” Network Analyzer MCODE plug‐in. Twenty clusters, characterized by highly associated domains, are obtained and visualized in Cytoscape (Table 6A). The cluster with the significantly higher score, Cluster 1, comprises 24 proteins and 218 interactions (edges) between these proteins (Figure 2). This cluster has nine primary circadian clock gene products and ribosomal proteins related to the circadian system.

**TABLE 6a:** MCODE clustering results for CPPIN2.

Cluster	Score	Nodes	Edges	Node IDs
1	7.478	24	218	KRR1, RPL21, CSNK1E, RPL3, NPAS2, EIF5B, RCL1, CSNK1D, RPS5, BMS1, PER1, WDR75, CLOCK, RPS25, WDR3, PER2, RPS23, CRY1, RPL14, NOP58, CRY2, RPL22, MPHOSPH10, ARNTL
2	5	5	23	ARID1B, SMARCB1, SMARCE1, BCL7B, SMARCC2
3	4.8	6	24	UBE2B, KLHL3, CUL3, UBE2C, ZBTB16, KLHL20
4	4	4	13	REPS2, REPS1, RALBP1, AP2S1
5	4	4	27	EXOSC2, EXOSC8, EXOSC7, EXOSC9
6	4	4	12	NUP98, NUP155, NUP107, NUP54
7	3.75	9	27	CTNNB1, TLE3, DVL3, TLE4, LDB1, AXIN1, HLX, LRP6, GSK3B
8	3.5	9	19	HSPA9, PPA2, GRPEL1, MRPS27, MRRF, LUC7L, TBRG4, LRPPRC, DNAJC19
9	3.333	4	8	LGALS9C, SLC12A6, TSPAN3, LGALS9
10	3.333	4	5	LARP4, FKBP8, AKAP1, GK
11	3.333	4	9	RAB5C, PACSIN2, PACSIN1, SYNJ1
12	3.333	4	10	LPAR2, FPR2, GNA11, GNG2
13	3.143	8	22	HNRNPA1, SNRPF, DDX42, TRA2B, KMT2C, HNRNPA3, RXRA, POLR2K
14	3	3	15	EIF3E, EIF3G, EIF3M
15	3	3	3	FKBP1B, TAGLN2, FABP5
16	3	3	6	SIN3A, PHF12, PHF21A
17	3	3	6	CACNA1C, CACNB2, CACNA2D3
18	3	3	6	MAPK1, ARRB1, QKI
19	3	3	7	USP14, PSMA3, PSMD8
20	3	3	3	GPATCH4, BRIX1, MAK16

To conduct a comprehensive analysis of APPIN5, it is also segmented into subclusters using the “Cytoscape” Network Analyzer MCODE plug‐in. A total of 16 clusters, characterized by highly associated domains, are extracted and visually represented using Cytoscape. The significant cluster, Cluster 1, comprises four proteins and a higher score in MCODE results. It includes the primary circadian clock gene products, ARNTL, ARNTL2, PER1, and NPAS2 (Table 7A). The identification of a cluster associated with circadian clock proteins within the pure autism network holds considerable significance. However, due to the small size of Cluster 1, it is not directly feasible to deduce a clear or significant connection between circadian clock proteins and ASD. Nevertheless, several studies (Ballester‐Navarro et al., 2021; Vallée et al., 2020; Yang et al., 2016) have provided insights indicating the interrelation between the circadian clock and autism. The thalamo‐cortico‐amygdala pathway, essential for complex emotional memory (Garcia et al., 2000), has been reported as dysfunctional in autism (Nicholas et al., 2007), these studies on neuronal PAS domain protein 2 (NPAS2) reveal that NPAS2‐deficient mice subjected to behavioral tests exhibit deficits in the long‐term memory. NPAS2 plays a regulatory role in acquiring specific types of memory, linking NPAS2 to autistic disorder. Moreover, a study (Nicholas et al., 2007) on the hypothesis that clock genes are implicated in autistic disorder revealed noteworthy associations in the PER1 and NPAS2 genes. Two single nucleotide polymorphisms (SNPs) within each gene reached conventional levels of statistical significance. Another significant finding emerges in APPIN5‐Cluster 16, encompassing SHANK2 and NLGN1 (Table 7A). Neurexin/neuroligin interactions in the neuronal synaptic cleft are crucial for the proper functioning of the synaptic network and neurotransmission (Südhof, 2008). A specific mRNA or translation process stimulates the local translation of significant ASD‐related proteins in the synaptic domain. Translation of the mRNAs for NLGN1, NLGN2, and NLGN3 is negatively regulated by Fragile X mental retardation protein (FMRP), loss of this translational repressor, FMRP, leads to Fragile X syndrome, associated with ASD (Joo & Benavides, 2021). Loss‐of‐function mutations in the NRXN1, NLGN3, and NLGN4 genes in ASD (Joo & Benavides, 2021; Tabuch et al., 2007) and disruption of SH3 and SHANK (multiple ankyrin repeat domains) lead to abnormal behaviors in ASD (Joo & Benavides, 2021; Monteiro & Feng, 2017). Some mutations in genes known to modulate synaptic plasticity are associated with rare cases of autism (Klauck, 2006). Therefore, the contribution of these synaptic plasticity‐related genetic factors to the development of abnormal central nervous system (CNS) in ASD requires further research.

**FIGURE 3:**
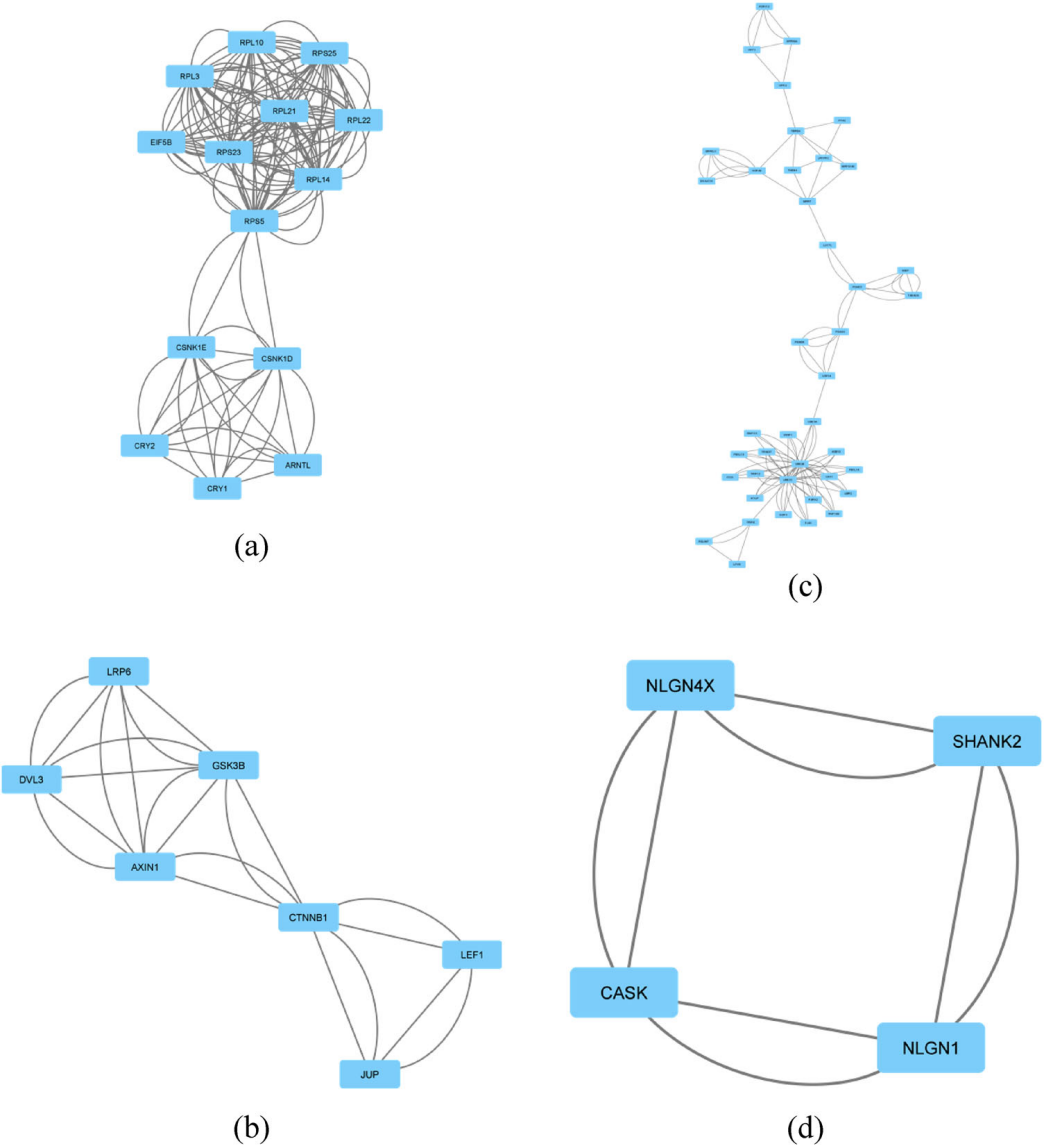
The interactome network combined circadian–autism protein–protein interaction network (CAPPIN) is subdivided into clusters of proteins with extensive and robust interactions. Clusters: (a) 1, (b) 10, (c) 12, (d) 30 extracted from Cytoscape.

To gain a deeper understanding of the combined network of circadian and autism‐related proteins, CAPPIN is divided into clusters using the “Cytoscape” Network Analyzer MCODE plug‐in. A total of 30 unique clusters are identified and visualized using Cytoscape (Table 8A). The highest scoring Cluster 1 (comprising 14 proteins and 145 edges) is included in the highest scoring cluster in CPPIN2. Within this combined network, seven additional clusters containing six or more proteins, are identified (Figure 3). Analyzing these clusters revealed a significant correlation between autism and circadian proteins involved in DNA and RNA transcription processes. The identification of Cluster 10, which is directly connected to the Wnt signaling pathway (Figure 3b), points out the importance of this pathway. The Wnt signaling pathway plays a crucial role in tumorigenesis (Zhan et al., 2017). Furthermore, numerous studies (Bae & Hong, 2018; Kwan et al., 2016; Zhang et al., 2014) have demonstrated the association between the Wnt signaling pathway and autism. The intensified transcription within the Wnt pathway is strongly correlated with the development of ASD (Mbadiwe & Millis, 2013). The binding of β‐catenin to the LEC/TCF influences the transcription of genes within the Wnt pathway (Mbadiwe & Millis, 2013). Upon examining Cluster 10, the CTNBB1 protein, which functions as the hub protein of the CAPPIN, plays crucial roles in the canonical Wnt signaling pathway and is intricately linked to autism (Kwan et al., 2016). CTNNB1 interacts with several ASD risk gene products within the APPINs. Nonsense and missense mutations in CTNNB1 have been identified in individuals with ASD. The CTNNB1 gene is recognized as a significant modulator among 22 ASD risk genes (O'Roak et al., 2012). GSK3B‐mediated Wnt signaling is implicated in amyloid peptide toxicity; however, inhibiting GSK3B activity might mitigate neurodegeneration (Schaffer et al., 2008).

### 3.4 Go enrichment analysis

The significant GO terms of the proteins within all clusters obtained through MCODE are identified using the GO Tool provided by Princeton University (Boyle et al., 2004). These enriched GO terms of the circadian proteins in CPPIN2‐Cluster 1 are presented along with their corresponding *p*‐values (Table 6A ). One of the molecular functions identified is blue light photoreceptor, which refers to a series of molecular signals triggered upon sensing of blue light by a photoreceptor molecule within a wavelength between a range of 400 and 470 nm (Tosini et al., 2016; Weber et al., 2019). The GO category of “blue light photoreceptor activity” exhibits an extremely low frequency within the genome, with only 2 out of 19,751 genes associated with it. Thus, the function of the blue light photoreceptor holds significance in comprehending the impact of light on the circadian clock, particularly given the correlation of the CRY1 and CRY2 genes with specific molecular functions. Additionally, other GO terms with higher cluster frequencies include transcription regulatory region sequence‐specific metabolic pathways. This term describes proteins that selectively and non‐covalently interact with specific sequences of DNA and RNA in regulatory regions, controlling transcription of sections of DNA and RNA molecules. The most significant finding is that the GO terms with high corrected *p*‐values are associated with circadian rhythm, glucocorticoid receptor modulation, ribosome biogenesis, and circadian control of gene expression (Table 6B).

**TABLE 6b:** Important gene ontology (GO) term functions of proteins in Cluster 1 in CPPIN2 including circadian main genes.

Gene ontology term	Cluster frequency	Genome frequency	Corrected *p*‐Value	Genes annotated to the term
Circadian regulation of gene expression	9 of 24 genes, 37.5%	66 of 19,751 genes, 0.3%	1.90e − 14	CSNK1E, ARNTL, CRY2, PER2, CSNK1D, NPAS2, CRY1, PER1, CLOCK
Negative regulation of glucocorticoid receptor signaling pathway	5 of 24 genes, 20.8%	6 of 19,751 genes, 0.0%	5.25e − 12	ARNTL, CRY2, CRY1, PER1, CLOCK
Gene expression	24 of 24 genes, 100.0%	5612 of 19,751 genes, 28.4%	3.81e − 11	RPL14, BMS1, CRY2, PER2, NOP58, WDR75, CSNK1D, MPHOSPH10, RPS5, WDR3, CSNK1E, RPL22, EIF5B, RPS25, RCL1, ARNTL, RPL21, NPAS2, RPL3, CRY1, PER1, KRR1, CLOCK, RPS23
Regulation of glucocorticoid receptor signaling pathway	5 of 24 genes, 20.8%	8 of 19,751 genes, 0.0%	4.89e − 11	ARNTL, CRY2, CRY1, PER1, CLOCK
Regulation of circadian rhythm	8 of 24 genes, 33.3%	123 of 19,751 genes, 0.6%	6.27e − 10	CSNK1E, ARNTL, CRY2, PER2, CSNK1D, CRY1, PER1, CLOCK
Ribosome biogenesis	10 of 24 genes, 41.7%	310 of 19,751 genes, 1.6%	6.54e − 10	WDR3, RPL14, RCL1, BMS1, NOP58, WDR75, RPL3, MPHOSPH10, KRR1, RPS5
Glucocorticoid receptor signaling pathway	5 of 24 genes, 20.8%	13 of 19,751 genes, 0.1%	1.12e − 09	ARNTL, CRY2, CRY1, PER1, CLOCK
Circadian rhythm	9 of 24 genes, 37.5%	220 of 19,751 genes, 1.1%	1.30e − 09	CSNK1E, ARNTL, CRY2, PER2, CSNK1D, NPAS2, CRY1, PER1, CLOCK
Ribonucleoprotein complex biogenesis	11 of 24 genes, 45.8%	483 of 19,751 genes, 2.4%	1.61e − 09	WDR3, RPL14, RCL1, BMS1, NOP58, WDR75, RPL3, MPHOSPH10, KRR1, RPS5, RPS23
Corticosteroid receptor signaling pathway	5 of 24 genes, 20.8%	14 of 19,751 genes, 0.1%	1.74e − 09	ARNTL, CRY2, CRY1, PER1, CLOCK
SRP‐dependent cotranslational protein targeting to membrane	7 of 24 genes, 29.2%	95 of 19,751 genes, 0.5%	7.94e − 09	RPL3, RPL22, RPL14, RPS25, RPS5, RPL21, RPS23
Cotranslational protein targeting to membrane	7 of 24 genes, 29.2%	100 of 19,751 genes, 0.5%	1.14e − 08	RPL3, RPL22, RPL14, RPS25, RPS5, RPL21, RPS23
Nucleic acid metabolic process	22 of 24 genes, 91.7%	5258 of 19,751 genes, 26.6%	1.73e − 08	RPL14, BMS1, CRY2, PER2, NOP58, WDR75, MPHOSPH10, RPS5, WDR3, CSNK1E, RPL22, RPS25, ARNTL, RCL1, RPL21, NPAS2, RPL3, CRY1, PER1, KRR1, CLOCK, RPS23
Protein targeting to ER	7 of 24 genes, 29.2%	110 of 19,751 genes, 0.6%	2.26e − 08	RPL3, RPL22, RPL14, RPS25, RPS5, RPL21, RPS23
Translational initiation	8 of 24 genes, 33.3%	193 of 19,751 genes, 1.0%	2.38e − 08	RPL22, EIF5B, RPL14, RPS25, RPL21, RPL3, RPS5, RPS23
Rhythmic process	9 of 24 genes, 37.5%	307 of 19,751 genes, 1.6%	2.59e − 08	CSNK1E, ARNTL, CRY2, PER2, CSNK1D, NPAS2, CRY1, PER1, CLOCK
Establishment of protein localization to endoplasmic reticulum	7 of 24 genes, 29.2%	114 of 19,751 genes, 0.6%	2.91e − 08	RPL3, RPL22, RPL14, RPS25, RPS5, RPL21, RPS23
RNA metabolic process	21 of 24 genes, 87.5%	4729 of 19,751 genes, 23.9%	4.26e − 08	RPL14, BMS1, CRY2, PER2, NOP58, WDR75, MPHOSPH10, RPS5, WDR3, RPL22, RPS25, ARNTL, RCL1, RPL21, NPAS2, RPL3, CRY1, PER1, KRR1, CLOCK, RPS23
Nuclear‐transcribed mRNA catabolic process, nonsense‐mediated decay	7 of 24 genes, 29.2%	121 of 19,751 genes, 0.6%	4.45e − 08	RPL3, RPL22, RPL14, RPS25, RPS5, RPL21, RPS23
rRNA processing	8 of 24 genes, 33.3%	227 of 19,751 genes, 1.1%	8.71e − 08	WDR3, RPL14, RCL1, BMS1, NOP58, WDR75, MPHOSPH10, KRR1
Cellular nitrogen compound metabolic process	23 of 24 genes, 95.8%	6649 of 19,751 genes, 33.7%	1.09e − 07	RPL14, BMS1, CRY2, PER2, NOP58, WDR75, MPHOSPH10, RPS5, WDR3, CSNK1E, RPL22, EIF5B, RPS25, ARNTL, RCL1, RPL21, NPAS2, RPL3, CRY1, PER1, KRR1, CLOCK, RPS23
Protein localization to endoplasmic reticulum	7 of 24 genes, 29.2%	141 of 19,751 genes, 0.7%	1.31e − 07	RPL3, RPL22, RPL14, RPS25, RPS5, RPL21, RPS23
Nucleobase‐containing compound metabolic process	22 of 24 genes, 91.7%	5936 of 19,751 genes, 30.1%	2.30e − 07	RPL14, BMS1, CRY2, PER2, NOP58, WDR75, MPHOSPH10, RPS5, WDR3, CSNK1E, RPL22, RPS25, ARNTL, RCL1, RPL21, NPAS2, RPL3, CRY1, PER1, KRR1, CLOCK, RPS23
Photoperiodism	5 of 24 genes, 20.8%	34 of 19,751 genes, 0.2%	2.38e − 07	CRY2, CRY1, PER2, PER1, CLOCK
rRNA metabolic process	8 of 24 genes, 33.3%	265 of 19,751 genes, 1.3%	2.97e − 07	WDR3, RPL14, RCL1, BMS1, NOP58, WDR75, MPHOSPH10, KRR1
Negative regulation of intracellular steroid hormone receptor signaling pathway	5 of 24 genes, 20.8%	36 of 19,751 genes, 0.2%	3.22e − 07	ARNTL, CRY2, CRY1, PER1, CLOCK
Heterocycle metabolic process	22 of 24 genes, 91.7%	6102 of 19,751 genes, 30.9%	4.13e − 07	RPL14, BMS1, CRY2, PER2, NOP58, WDR75, MPHOSPH10, RPS5, WDR3, CSNK1E, RPL22, RPS25, ARNTL, RCL1, RPL21, NPAS2, RPL3, CRY1, PER1, KRR1, CLOCK, RPS23
Cellular aromatic compound metabolic process	22 of 24 genes, 91.7%	6151 of 19,751 genes, 31.1%	4.89e − 07	RPL14, BMS1, CRY2, PER2, NOP58, WDR75, MPHOSPH10, RPS5, WDR3, CSNK1E, RPL22, RPS25, ARNTL, RCL1, RPL21, NPAS2, RPL3, CRY1, PER1, KRR1, CLOCK, RPS23
Viral transcription	7 of 24 genes, 29.2%	178 of 19,751 genes, 0.9%	6.73e − 07	RPL3, RPL22, RPL14, RPS25, RPS5, RPL21, RPS23
Organic cyclic compound metabolic process	22 of 24 genes, 91.7%	6369 of 19,751 genes, 32.2%	1.02e − 06	RPL14, BMS1, CRY2, PER2, NOP58, WDR75, MPHOSPH10, RPS5, WDR3, CSNK1E, RPL22, RPS25, ARNTL, RCL1, RPL21, NPAS2, RPL3, CRY1, PER1, KRR1, CLOCK, RPS23
Translation	10 of 24 genes, 41.7%	658 of 19,751 genes, 3.3%	1.04e − 06	RPL22, EIF5B, RPL14, RPS25, PER2, RPL21, RPL3, PER1, RPS5, RPS23
Viral gene expression	7 of 24 genes, 29.2%	195 of 19,751 genes, 1.0%	1.27e − 06	RPL3, RPL22, RPL14, RPS25, RPS5, RPL21, RPS23
Peptide biosynthetic process	10 of 24 genes, 41.7%	683 of 19,751 genes, 3.5%	1.49e − 06	RPL22, EIF5B, RPL14, RPS25, PER2, RPL21, RPL3, PER1, RPS5, RPS23
Protein targeting to membrane	7 of 24 genes, 29.2%	202 of 19,751 genes, 1.0%	1.62e − 06	RPL3, RPL22, RPL14, RPS25, RPS5, RPL21, RPS23
Nuclear‐transcribed mRNA catabolic process	7 of 24 genes, 29.2%	211 of 19,751 genes, 1.1%	2.20e − 06	RPL3, RPL22, RPL14, RPS25, RPS5, RPL21, RPS23
ncRNA processing	8 of 24 genes, 33.3%	400 of 19,751 genes, 2.0%	7.54e − 06	WDR3, RPL14, RCL1, BMS1, NOP58, WDR75, MPHOSPH10, KRR1
Amide biosynthetic process	10 of 24 genes, 41.7%	828 of 19,751 genes, 4.2%	9.41e − 06	RPL22, EIF5B, RPL14, RPS25, PER2, RPL21, RPL3, PER1, RPS5, RPS23
Peptide metabolic process	10 of 24 genes, 41.7%	841 of 19,751 genes, 4.3%	1.09e − 05	RPL22, EIF5B, RPL14, RPS25, PER2, RPL21, RPL3, PER1, RPS5, RPS23
Macromolecule metabolic process	24 of 24 genes, 100.0%	9565 of 19,751 genes, 48.4%	1.40e − 05	RPL14, BMS1, CRY2, PER2, NOP58, WDR75, CSNK1D, MPHOSPH10, RPS5, WDR3, CSNK1E, RPL22, EIF5B, RPS25, RCL1, ARNTL, RPL21, NPAS2, RPL3, CRY1, PER1, KRR1, CLOCK, RPS23
Regulation of intracellular steroid hormone receptor signaling pathway	5 of 24 genes, 20.8%	80 of 19,751 genes, 0.4%	1.98e − 05	ARNTL, CRY2, CRY1, PER1, CLOCK
Entrainment of circadian clock by photoperiod	4 of 24 genes, 16.7%	30 of 19,751 genes, 0.2%	2.32e − 05	CRY2, CRY1, PER2, PER1
Cellular macromolecule catabolic process	11 of 24 genes, 45.8%	1204 of 19,751 genes, 6.1%	2.54e − 05	RPL22, CSNK1E, RPL14, RPS25, ARNTL, RPL21, CSNK1D, RPL3, RPS5, CLOCK, RPS23
Entrainment of circadian clock	4 of 24 genes, 16.7%	35 of 19,751 genes, 0.2%	4.41e − 05	CRY2, CRY1, PER2, PER1
Negative regulation of gene expression	13 of 24 genes, 54.2%	1984 of 19,751 genes, 10.0%	4.53e − 05	RPL22, RPL14, RPS25, ARNTL, CRY2, PER2, RPL21, RPL3, CRY1, RPS5, PER1, CLOCK, RPS23
Establishment of protein localization to membrane	7 of 24 genes, 29.2%	335 of 19,751 genes, 1.7%	5.28e − 05	RPL3, RPL22, RPL14, RPS25, RPS5, RPL21, RPS23
Regulation of gene expression	18 of 24 genes, 75.0%	4660 of 19,751 genes, 23.6%	7.68e − 05	RPL14, CRY2, PER2, WDR75, CSNK1D, RPS5, CSNK1E, RPL22, EIF5B, RPS25, ARNTL, RPL21, NPAS2, RPL3, CRY1, PER1, CLOCK, RPS23
Nitrogen compound metabolic process	24 of 24 genes, 100.0%	10,332 of 19,751 genes, 52.3%	8.98e − 05	RPL14, BMS1, CRY2, PER2, NOP58, WDR75, CSNK1D, MPHOSPH10, RPS5, WDR3, CSNK1E, RPL22, EIF5B, RPS25, RCL1, ARNTL, RPL21, NPAS2, RPL3, CRY1, PER1, KRR1, CLOCK, RPS23
Establishment of protein localization to organelle	8 of 24 genes, 33.3%	552 of 19,751 genes, 2.8%	9.04e − 05	RPL22, RPL14, RPS25, ARNTL, RPL21, RPL3, RPS5, RPS23
mRNA catabolic process	7 of 24 genes, 29.2%	377 of 19,751 genes, 1.9%	0.00011	RPL3, RPL22, RPL14, RPS25, RPS5, RPL21, RPS23
ncRNA metabolic process	8 of 24 genes, 33.3%	586 of 19,751 genes, 3.0%	0.00014	WDR3, RPL14, RCL1, BMS1, NOP58, WDR75, MPHOSPH10, KRR1
Intracellular steroid hormone receptor signaling pathway	5 of 24 genes, 20.8%	119 of 19,751 genes, 0.6%	0.00014	ARNTL, CRY2, CRY1, PER1, CLOCK
Macromolecule catabolic process	11 of 24 genes, 45.8%	1438 of 19,751 genes, 7.3%	0.00015	RPL22, CSNK1E, RPL14, RPS25, ARNTL, RPL21, CSNK1D, RPL3, RPS5, CLOCK, RPS23
Cellular amide metabolic process	10 of 24 genes, 41.7%	1118 of 19,751 genes, 5.7%	0.00015	RPL22, EIF5B, RPL14, RPS25, PER2, RPL21, RPL3, PER1, RPS5, RPS23
RNA catabolic process	7 of 24 genes, 29.2%	417 of 19,751 genes, 2.1%	0.00023	RPL3, RPL22, RPL14, RPS25, RPS5, RPL21, RPS23
Primary metabolic process	24 of 24 genes, 100.0%	10,810 of 19,751 genes, 54.7%	0.00026	RPL14, BMS1, CRY2, PER2, NOP58, WDR75, CSNK1D, MPHOSPH10, RPS5, WDR3, CSNK1E, RPL22, EIF5B, RPS25, RCL1, ARNTL, RPL21, NPAS2, RPL3, CRY1, PER1, KRR1, CLOCK, RPS23
Cellular metabolic process	24 of 24 genes, 100.0%	10,825 of 19,751 genes, 54.8%	0.00027	RPL14, BMS1, CRY2, PER2, NOP58, WDR75, CSNK1D, MPHOSPH10, RPS5, WDR3, CSNK1E, RPL22, EIF5B, RPS25, RCL1, ARNTL, RPL21, NPAS2, RPL3, CRY1, PER1, KRR1, CLOCK, RPS23
Response to redox state	3 of 24 genes, 12.5%	14 of 19,751 genes, 0.1%	0.00029	NPAS2, ARNTL, CLOCK
Protein targeting	7 of 24 genes, 29.2%	436 of 19,751 genes, 2.2%	0.00031	RPL3, RPL22, RPL14, RPS25, RPS5, RPL21, RPS23
Steroid hormone–mediated signaling pathway	5 of 24 genes, 20.8%	142 of 19,751 genes, 0.7%	0.00035	ARNTL, CRY2, CRY1, PER1, CLOCK
Negative regulation of circadian rhythm	3 of 24 genes, 12.5%	15 of 19,751 genes, 0.1%	0.00036	CRY2, CRY1, PER2
Protein localization to organelle	9 of 24 genes, 37.5%	946 of 19,751 genes, 4.8%	0.00044	RPL22, RPL14, RPS25, ARNTL, RPL21, CSNK1D, RPL3, RPS5, RPS23
Organic substance metabolic process	24 of 24 genes, 100.0%	11,184 of 19,751 genes, 56.6%	0.00060	RPL14, BMS1, CRY2, PER2, NOP58, WDR75, CSNK1D, MPHOSPH10, RPS5, WDR3, CSNK1E, RPL22, EIF5B, RPS25, RCL1, ARNTL, RPL21, NPAS2, RPL3, CRY1, PER1, KRR1, CLOCK, RPS23
Response to blue light	2 of 24 genes, 8.3%	2 of 19,751 genes, 0.0%	0.00073	CRY2, CRY1
Blue light–signaling pathway	2 of 24 genes, 8.3%	2 of 19,751 genes, 0.0%	0.00073	CRY2, CRY1
Cellular response to blue light	2 of 24 genes, 8.3%	2 of 19,751 genes, 0.0%	0.00073	CRY2, CRY1
Regulation of Wnt‐mediated midbrain dopaminergic neuron differentiation	2 of 24 genes, 8.3%	2 of 19,751 genes, 0.0%	0.00073	CSNK1E, CSNK1D
Positive regulation of Wnt‐mediated midbrain dopaminergic neuron differentiation	2 of 24 genes, 8.3%	2 of 19,751 genes, 0.0%	0.00073	CSNK1E, CSNK1D
Ribosomal small subunit biogenesis	4 of 24 genes, 16.7%	75 of 19,751 genes, 0.4%	0.00099	WDR3, RCL1, BMS1, RPS5
Negative regulation of metabolic process	14 of 24 genes, 58.3%	3098 of 19,751 genes, 15.7%	0.00112	RPL22, RPL14, RPS25, ARNTL, CRY2, PER2, RPL21, RPL3, MPHOSPH10, CRY1, RPS5, PER1, CLOCK, RPS23
Macromolecule localization	14 of 24 genes, 58.3%	3121 of 19,751 genes, 15.8%	0.00122	RPL22, CSNK1E, RPL14, RPS25, ARNTL, PER2, NOP58, RPL21, CSNK1D, RPL3, CRY1, RPS5, CLOCK, RPS23
Metabolic process	24 of 24 genes, 100.0%	11,677 of 19,751 genes, 59.1%	0.00169	RPL14, BMS1, CRY2, PER2, NOP58, WDR75, CSNK1D, MPHOSPH10, RPS5, WDR3, CSNK1E, RPL22, EIF5B, RPS25, RCL1, ARNTL, RPL21, NPAS2, RPL3, CRY1, PER1, KRR1, CLOCK, RPS23
Hormone‐mediated signaling pathway	5 of 24 genes, 20.8%	202 of 19,751 genes, 1.0%	0.00199	ARNTL, CRY2, CRY1, PER1, CLOCK
Positive regulation of midbrain dopaminergic neuron differentiation	2 of 24 genes, 8.3%	3 of 19,751 genes, 0.0%	0.00218	CSNK1E, CSNK1D
Cellular response to steroid hormone stimulus	5 of 24 genes, 20.8%	209 of 19,751 genes, 1.1%	0.00235	ARNTL, CRY2, CRY1, PER1, CLOCK
Regulation of hair cycle	3 of 24 genes, 12.5%	29 of 19,751 genes, 0.1%	0.00291	ARNTL, PER1, CLOCK
Negative regulation of macromolecule metabolic process	13 of 24 genes, 54.2%	2845 of 19,751 genes, 14.4%	0.00301	RPL22, RPL14, RPS25, ARNTL, CRY2, PER2, RPL21, RPL3, CRY1, RPS5, PER1, CLOCK, RPS23
Protein localization to membrane	7 of 24 genes, 29.2%	645 of 19,751 genes, 3.3%	0.00421	RPL3, RPL22, RPL14, RPS25, RPS5, RPL21, RPS23
Circadian regulation of translation	2 of 24 genes, 8.3%	4 of 19,751 genes, 0.0%	0.00437	PER2, PER1
Positive regulation of dopaminergic neuron differentiation	2 of 24 genes, 8.3%	4 of 19,751 genes, 0.0%	0.00437	CSNK1E, CSNK1D
Nucleobase‐containing compound catabolic process	7 of 24 genes, 29.2%	673 of 19,751 genes, 3.4%	0.00556	RPL3, RPL22, RPL14, RPS25, RPS5, RPL21, RPS23
RNA processing	8 of 24 genes, 33.3%	959 of 19,751 genes, 4.9%	0.00567	WDR3, RPL14, RCL1, BMS1, NOP58, WDR75, MPHOSPH10, KRR1
Regulation of metabolic process	19 of 24 genes, 79.2%	6910 of 19,751 genes, 35.0%	0.00624	RPL14, CRY2, PER2, WDR75, CSNK1D, MPHOSPH10, RPS5, CSNK1E, RPL22, EIF5B, RPS25, ARNTL, RPL21, NPAS2, RPL3, CRY1, PER1, CLOCK, RPS23
Regulation of midbrain dopaminergic neuron differentiation	2 of 24 genes, 8.3%	5 of 19,751 genes, 0.0%	0.00728	CSNK1E, CSNK1D
Intracellular receptor signaling pathway	5 of 24 genes, 20.8%	267 of 19,751 genes, 1.4%	0.00772	ARNTL, CRY2, CRY1, PER1, CLOCK
Heterocycle catabolic process	7 of 24 genes, 29.2%	720 of 19,751 genes, 3.6%	0.00862	RPL3, RPL22, RPL14, RPS25, RPS5, RPL21, RPS23
Cellular nitrogen compound catabolic process	7 of 24 genes, 29.2%	721 of 19,751 genes, 3.7%	0.00870	RPL3, RPL22, RPL14, RPS25, RPS5, RPL21, RPS23

**TABLE 7a:** MCODE clustering results for APPIN5.

Cluster	Score	Nodes	Edges	Node IDs
1	4	4	10	ARNTL2, PER1, ARNTL, NPAS2
2	4	4	16	SEPT1, SEPT2, SEPT6, SEPT5
3	4	4	12	JUP, APC, TCF7L2, CTNNB1
4	3.333	4	15	MYOG, TCF12, LDB1, TCF4
5	3.333	4	12	AP2S1, NTRK1, HGS, AP2B1
6	3.333	4	19	PRKAA2, PRKAG1, PRKAB2, PRKAA1
7	3.333	4	7	NDE1, NUP54, NDEL1, SNAP29
8	3	3	6	DLG2, NLGN3, NRXN1
9	3	3	3	IKZF3, GATAD2B, IKZF1
10	3	3	4	TRIP6, LPXN, PDLIM7
11	3	3	8	MBIP, TADA2A, PSME3
12	3	3	6	PHF21A, PHF12, SIN3A
13	3	3	5	RBCK1, RABGEF1, RNF31
14	3	3	6	NRG1, NRG3, ERBB2
15	3	3	6	HRAS, ITGB3, MAPK3
16	2.667	4	8	NLGN4X, CASK, SHANK2, NLGN1

The significant GO terms of the proteins in Clusters 2 and 16 of APPIN5 are tabulated with *p*‐values in Table 7B and C. The enriched GO terms for Cluster 2 are mainly related to circadian rhythm and its regulation, as well as the redox state. Cluster 16 has enriched GO terms associated with the regulation of transcription by RNA polymerase II, regulation of RNA biosynthetic process, RNA metabolic process, regulation of nitrogen compound metabolic process, canonical Wnt signaling pathway involved in regulation, and more.

**TABLE 7b:** Important gene ontology (GO) term functions of proteins in Cluster 1 in APPIN5 including circadian main genes.

Gene ontology term	Cluster frequency	Genome frequency	Corrected *p*‐Value	Genes annotated to the term
Circadian regulation of gene expression	4 of 4 genes, 100.0%	69 of 19,810 genes, 0.3%	2.08e − 08	NPAS2, ARNTL, PER1, ARNTL2
Circadian rhythm	4 of 4 genes, 100.0%	207 of 19,810 genes, 1.0%	1.79e − 06	NPAS2, ARNTL, PER1, ARNTL2
Rhythmic process	4 of 4 genes, 100.0%	300 of 19,810 genes, 1.5%	7.99e − 06	NPAS2, ARNTL, PER1, ARNTL2
Negative regulation of glucocorticoid receptor signaling pathway	2 of 4 genes, 50.0%	6 of 19,810 genes, 0.0%	0.000071	ARNTL, PER1
Regulation of glucocorticoid receptor signaling pathway	2 of 4 genes, 50.0%	7 of 19,810 genes, 0.0%	9.95e − 05	ARNTL, PER1
Regulation of circadian rhythm	3 of 4 genes, 75.0%	118 of 19,810 genes, 0.6%	0.00012	ARNTL, PER1, ARNTL2
Glucocorticoid receptor signaling pathway	2 of 4 genes, 50.0%	13 of 19,810 genes, 0.1%	0.00036	ARNTL, PER1
Response to redox state	2 of 4 genes, 50.0%	13 of 19,810 genes, 0.1%	0.00036	NPAS2, ARNTL
Corticosteroid receptor signaling pathway	2 of 4 genes, 50.0%	14 of 19,810 genes, 0.1%	0.00043	ARNTL, PER1
Positive regulation of circadian rhythm	2 of 4 genes, 50.0%	17 of 19,810 genes, 0.1%	0.00064	ARNTL, ARNTL2
Regulation of hair cycle	2 of 4 genes, 50.0%	28 of 19,810 genes, 0.1%	0.00178	ARNTL, PER1
Positive regulation of transcription by RNA polymerase II	4 of 4 genes, 100.0%	1200 of 19,810 genes, 6.1%	0.00207	NPAS2, ARNTL, PER1, ARNTL2
Entrainment of circadian clock	2 of 4 genes, 50.0%	35 of 19,810 genes, 0.2%	0.00281	PER1, ARNTL2
Negative regulation of intracellular steroid hormone receptor signaling pathway	2 of 4 genes, 50.0%	38 of 19,810 genes, 0.2%	0.00332	ARNTL, PER1
Positive regulation of transcription, DNA‐templated	4 of 4 genes, 100.0%	1616 of 19,810 genes, 8.2%	0.00684	NPAS2, ARNTL, PER1, ARNTL2
Positive regulation of nucleic acid‐templated transcription	4 of 4 genes, 100.0%	1616 of 19,810 genes, 8.2%	0.00684	NPAS2, ARNTL, PER1, ARNTL2
Positive regulation of RNA biosynthetic process	4 of 4 genes, 100.0%	1622 of 19,810 genes, 8.2%	0.00694	NPAS2, ARNTL, PER1, ARNTL2
Positive regulation of RNA metabolic process	4 of 4 genes, 100.0%	1750 of 19,810 genes, 8.8%	0.0094	NPAS2, ARNTL, PER1, ARNTL2

**TABLE 8a:** MCODE clustering results for combined circadian–autism protein–protein interaction network (CAPPIN).

Cluster	Score	Nodes	Edges	Node IDs
1	7.385	14	145	CSNK1D, RPS25, RPL10, RPS23, RPL14, RPL22, CRY2, CRY1, RPL21, RPL3, ARNTL, EIF5B, CSNK1E, RPS5
2	7	7	42	RCL1, NOP58, BMS1, MPHOSPH10, KRR1, WDR75, WDR3
3	6.857	8	49	SNRPN, HNRNPA3, DDX42, HNRNPA1, TRA2B, PPIL1, SNRPF, PPIL4
4	5	5	23	BCL7B, SMARCC2, SMARCE1, SMARCB1, ARID1B
5	5	5	21	NUP54, NUP98, NUP62, NUP155, NUP107
6	4.8	6	24	QKI, ARRB1, HRAS, MAPK3, ITGB3, MAPK1
7	4	4	16	SEPT6, SEPT5, SEPT1, SEPT2
8	4	4	17	PRKAB2, PRKAA1, MCL1, PRKAG1
9	4	4	27	EXOSC8, EXOSC2, EXOSC7, EXOSC9
10	3.667	7	22	AXIN1, CTNNB1, DVL3, GSK3B, LRP6, LEF1, JUP
11	3.6	6	9	GPATCH4, MYBBP1A, BRIX1, DGCR8, MAK16, MECP2
12	3.4	41	119	DZIP3, THEM4, PPA2, DNAJC19, PJA1, HSPA9, ASB13, GRPEL1, MYLIP, LPXN, TRIP6, USP14, VIPR2, ITCH, YIPF3, PSMA3, RNF111, FBXL13, PSMD8, TADA2A, UBE3A, LRPPRC, MBIP, WWP1, MRPS18C, PSME3, MRRF, UBE2C, GPR89A, FBXL16, P2RY12, TBRG4, UBE2B, TRIP12, LNX1, RNF19B, LUC7L, UBR2, PDLIM7, PARK2, TRIM37
13	3.333	4	10	TLE3, TLE4, AES, LDB1
14	3.333	4	5	JUNB, RFWD2, TRIB2, TRIB1
15	3.333	4	8	LGALS9, LGALS9C, SLC12A6, TSPAN3
16	3.333	4	5	FKBP8, GK, LARP4, AKAP1
17	3.333	4	10	CACNB2, CACNA1C, CACNA2D3, CACNA1F
18	3	3	15	EIF3E, EIF3G, EIF3M
19	3	3	6	NLGN3, NRXN1, DLG2
20	3	3	7	RALBP1, REPS1, REPS2
21	3	3	6	EPS15L1, OCRL, HGS
22	3	3	8	AP2B1, AP2S1, NTRK1
23	3	3	3	IKZF3, IKZF1, GATAD2B
24	3	3	3	FABP5, FKBP1B, TAGLN2
25	3	3	6	SIN3A, PHF12, PHF21A
26	3	3	5	TRAF2, TRAF6, IRF7
27	3	3	3	IKBIP, FBXO28, TRAF7
28	3	3	5	NDEL1, SNAP29, NDE1
29	2.667	4	8	CLOCK, PER1, NPAS2, PER2
30	2.667	4	8	SHANK2, NLGN1, CASK, NLGN4X

The enriched GO terms for CAPPIN Cluster 1 primarily pertain to cytoplasmic translation or translation, glucocorticoid receptor signaling pathway and its regulation, ribonucleoprotein complex biogenesis, cellular response to endogenous stimulus, histone acetylation, and peptidyl‐lysine acetylation processes (Table 8B). MAPK3 (present in APPIN and CAPPIN) and RPS6KA4 (present in CPPIN2 and CAPPIN) are involved in the positive regulation of peptidyl‐lysine acetylation and have been found to be influenced by antidepressants and related molecular pathway analogs (Drago et al., 2011). The enriched GO terms for CAPPIN Cluster 10 are primarily related to Wnt signaling pathway, regulation of macromolecule biosynthetic process, regulation of RNA biosynthetic process, and midbrain dopaminergic neuron differentiation.

**TABLE 8b:** Important gene ontology (GO) terms of the proteins in Cluster 1 of combined circadian–autism protein–protein interaction network (CAPPIN) obtained using the Gene Ontology Tool developed by Princeton University (Boyle, et al.,^2004^).

Gene ontology term	Genes annotated to the term
Cytoplasmic translation	RPL22, RPL14, RPS25, RPL10, RPL21, RPL3, RPS5, RPS23
Translation	RPL22, EIF5B, RPL14, RPS25, RPL10, RPL21, RPL3, RPS5, RPS23
Peptide biosynthetic process	RPL22, EIF5B, RPL14, RPS25, RPL10, RPL21, RPL3, RPS5, RPS23
Amide biosynthetic process	RPL22, EIF5B, RPL14, RPS25, RPL10, RPL21, RPL3, RPS5, RPS23
Peptide metabolic process	RPL22, EIF5B, RPL14, RPS25, RPL10, RPL21, RPL3, RPS5, RPS23
Circadian regulation of gene expression	CSNK1E, ARNTL, CRY1, CRY2, CSNK1D
Cellular protein metabolic process	CSNK1E, RPL22, EIF5B, RPL14, ARNTL, RPS25, CRY2, RPL10, RPL21, CSNK1D, RPL3, CRY1, RPS5, RPS23
Negative regulation of glucocorticoid receptor signaling pathway	ARNTL, CRY1, CRY2
Cellular amide metabolic process	RPL22, EIF5B, RPL14, RPS25, RPL10, RPL21, RPL3, RPS5, RPS23
Regulation of glucocorticoid receptor signaling pathway	ARNTL, CRY1, CRY2
Protein metabolic process	CSNK1E, RPL22, EIF5B, RPL14, ARNTL, RPS25, CRY2, RPL10, RPL21, CSNK1D, RPL3, CRY1, RPS5, RPS23
Regulation of circadian rhythm	CSNK1E, ARNTL, CRY1, CRY2, CSNK1D
Gene expression	CSNK1E, RPL22, EIF5B, RPL14, ARNTL, RPS25, CRY2, RPL10, RPL21, CSNK1D, RPL3, CRY1, RPS5, RPS23
Glucocorticoid receptor signaling pathway	ARNTL, CRY1, CRY2
Corticosteroid receptor signaling pathway	ARNTL, CRY1, CRY2
Organonitrogen compound metabolic process	CSNK1E, RPL22, EIF5B, RPL14, ARNTL, RPS25, CRY2, RPL10, RPL21, CSNK1D, RPL3, CRY1, RPS5, RPS23
Circadian rhythm	CSNK1E, ARNTL, CRY1, CRY2, CSNK1D
Organonitrogen compound biosynthetic process	RPL22, EIF5B, RPL14, RPS25, RPL10, RPL21, RPL3, RPS5, RPS23
Blue light signaling pathway	CRY1, CRY2
Cellular response to blue light	CRY1, CRY2
Regulation of Wnt‐mediated midbrain dopaminergic neuron differentiation	CSNK1E, CSNK1D
Positive regulation of Wnt‐mediated midbrain dopaminergic neuron differentiation	CSNK1E, CSNK1D
Rhythmic process	CSNK1E, ARNTL, CRY1, CRY2, CSNK1D
Positive regulation of midbrain dopaminergic neuron differentiation	CSNK1E, CSNK1D
Negative regulation of steroid hormone secretion	CRY1, CRY2
Negative regulation of corticosteroid hormone secretion	CRY1, CRY2
Negative regulation of glucocorticoid secretion	CRY1, CRY2
Ribosome biogenesis	RPL3, RPL14, RPS25, RPS5, RPL10
Cellular nitrogen compound biosynthetic process	RPL22, EIF5B, RPL14, ARNTL, RPS25, CRY2, RPL10, RPL21, RPL3, CRY1, RPS5, RPS23
Cellular macromolecule biosynthetic process	RPL22, EIF5B, RPL14, ARNTL, RPS25, CRY2, RPL10, RPL21, RPL3, CRY1, RPS5, RPS23
Macromolecule biosynthetic process	RPL22, EIF5B, RPL14, ARNTL, RPS25, CRY2, RPL10, RPL21, RPL3, CRY1, RPS5, RPS23
Negative regulation of intracellular steroid hormone receptor signaling pathway	ARNTL, CRY1, CRY2
Cellular macromolecule metabolic process	CSNK1E, RPL22, EIF5B, RPL14, ARNTL, RPS25, CRY2, RPL10, RPL21, CSNK1D, RPL3, CRY1, RPS5, RPS23
Positive regulation of dopaminergic neuron differentiation	CSNK1E, CSNK1D
Cellular nitrogen compound metabolic process	CSNK1E, RPL22, EIF5B, RPL14, ARNTL, RPS25, CRY2, RPL10, RPL21, RPL3, CRY1, RPS5, RPS23
Regulation of midbrain dopaminergic neuron differentiation	CSNK1E, CSNK1D
Non‐membrane‐bounded organelle assembly	RPL3, RPS5, RPL10, RPS23, CSNK1D
Response to blue light	CRY1, CRY2
Ribonucleoprotein complex biogenesis	RPL3, RPL14, RPS25, RPS5, RPL10
Ribosome assembly	RPL3, RPS5, RPL10
Cellular biosynthetic process	RPL22, EIF5B, RPL14, ARNTL, RPS25, CRY2, RPL10, RPL21, RPL3, CRY1, RPS5, RPS23
Regulation of glucocorticoid secretion	CRY1, CRY2
Ribosomal large subunit biogenesis	RPL3, RPL14, RPL10
Regulation of intracellular steroid hormone receptor signaling pathway	ARNTL, CRY1, CRY2
Organic substance biosynthetic process	RPL22, EIF5B, RPL14, ARNTL, RPS25, CRY2, RPL10, RPL21, RPL3, CRY1, RPS5, RPS23
Regulation of dopaminergic neuron differentiation	CSNK1E, CSNK1D
Biosynthetic process	RPL22, EIF5B, RPL14, ARNTL, RPS25, CRY2, RPL10, RPL21, RPL3, CRY1, RPS5, RPS23
Glucocorticoid secretion	CRY1, CRY2
Macromolecule metabolic process	CSNK1E, RPL22, EIF5B, RPL14, ARNTL, RPS25, CRY2, RPL10, RPL21, CSNK1D, RPL3, CRY1, RPS5, RPS23

Indeed, there are 49 shared proteins present in CPPIN2 and APPIN5. The enriched GO terms associated with these proteins primarily revolve around regulation processes, including the regulation of transcription by RNA polymerase II, regulation of RNA metabolic process, regulation of transcription, DNA‐templated, regulation of transport, regulation of localization, regulation of ion transport, histone modification and acetylation, peptidyl‐lysine modification and acetylation, as well as multicellular organism development (Table 9). The significant role of histone acetylation in the context of ASD has recently been elucidated and warrants further in‐depth investigation. Alterations in histone acetylation have the potential to induce modifications in gene transcription, playing a crucial role in the development of ASD. Notably, the mRNA levels of ASD‐related risk genes like NLGN1, SHANK2, SHANK3, and CNTNAP2 are influenced by the prenatal suppression of histone deacetylase family, which are linked to neurodevelopmental disorders (Tseng et al., 2022). Furthermore, prenatal exposure to environmental factors has the potential to induce modifications in epigenetic markers within utero through various mechanisms, including chemical modifications like DNA methylation or protein modifications such as histone acetylation/deacetylation. These changes have been implicated in contributing to neurodevelopmental consequences associated with ASD (Tseng et al., 2022).

**TABLE 9:** Enrichment analysis performed on 49 common proteins in CPPIN2 and APPIN5 obtained using the Gene Ontology Tool developed by Princeton University (Boyle et al., 2004).

Gene ontology term	Genes annotated to the term
Regulation of transcription by RNA polymerase II	CTBP1, TCF4, ESR1, SIN3A, SMARCC2, LDB1, PHF21A, CHD8, TBL1XR1, FOXP2, FOXP1, CTNNB1, MKX, ARNTL, PHF12, MED13L, NPAS2, TSC22D4, KMT2C, ARID1B, NCOA1, TCF7L2, PER1, CREBBP
Transcription by RNA polymerase II	CTBP1, TCF4, ESR1, SIN3A, SMARCC2, LDB1, PHF21A, CHD8, TBL1XR1, FOXP2, FOXP1, CTNNB1, MKX, ARNTL, PHF12, MED13L, NPAS2, TSC22D4, KMT2C, ARID1B, NCOA1, TCF7L2, PER1, CREBBP
Negative regulation of transcription, DNA‐templated	CTBP1, ESR1, SIN3A, SMARCC2, LDB1, PHF21A, CHD8, TBL1XR1, FOXP2, FOXP1, CTNNB1, ARNTL, PHF12, TSC22D4, TCF7L2, PER1, CREBBP
Negative regulation of nucleic acid‐templated transcription	CTBP1, ESR1, SIN3A, SMARCC2, LDB1, PHF21A, CHD8, TBL1XR1, FOXP2, FOXP1, CTNNB1, ARNTL, PHF12, TSC22D4, TCF7L2, PER1, CREBBP
Negative regulation of RNA biosynthetic process	CTBP1, ESR1, SIN3A, SMARCC2, LDB1, PHF21A, CHD8, TBL1XR1, FOXP2, FOXP1, CTNNB1, ARNTL, PHF12, TSC22D4, TCF7L2, PER1, CREBBP
Negative regulation of RNA metabolic process	CTBP1, ESR1, SIN3A, SMARCC2, LDB1, PHF21A, CHD8, TBL1XR1, FOXP2, FOXP1, CTNNB1, ARNTL, PHF12, TSC22D4, TCF7L2, PER1, CREBBP
Regulation of biological process	CTBP1, TCF4, SIN3A, SMARCC2, GNAI1, GFAP, CACNA1C, PPP2R5D, PHF21A, DYNC1H1, UBR1, FOXP2, GABRB3, FOXP1, GABRB2, MKX, TRAF7, NUP155, ANK2, KMT2C, NCOA1, CORO1A, PER1, ESR1, PPP5C, LDB1, NDEL1, CHD8, TBL1XR1, CACNA1E, STXBP1, CACNA2D3, CTNNB1, ARNTL, NRCAM, PHF12, MED13L, SCN2A, NPAS2, TSC22D4, ARID1B, TCF7L2, SCN1A, NUP54, CREBBP, HTR1B
Negative regulation of transcription by RNA polymerase II	CTBP1, ESR1, CTNNB1, SIN3A, LDB1, PHF12, PHF21A, CHD8, TBL1XR1, FOXP2, TCF7L2, PER1, FOXP1, CREBBP
Biological regulation	CTBP1, TCF4, SIN3A, STX11, SMARCC2, GNAI1, GFAP, CACNA1C, PPP2R5D, PHF21A, DYNC1H1, UBR1, FOXP2, GABRB3, FOXP1, GABRB2, MKX, TRAF7, NUP155, ANK2, KMT2C, NCOA1, CORO1A, PER1, ESR1, PPP5C, LDB1, NDEL1, CHD8, TBL1XR1, CACNA1E, STXBP1, CACNA2D3, CTNNB1, ARNTL, NRCAM, PHF12, MED13L, SCN2A, NPAS2, TSC22D4, ARID1B, TCF7L2, SCN1A, NUP54, CREBBP, HTR1B
Negative regulation of nucleobase‐containing compound metabolic process	CTBP1, ESR1, SIN3A, SMARCC2, LDB1, PHF21A, CHD8, TBL1XR1, FOXP2, FOXP1, CTNNB1, ARNTL, PHF12, TSC22D4, TCF7L2, PER1, CREBBP
Regulation of cellular process	CTBP1, TCF4, SIN3A, SMARCC2, GNAI1, GFAP, CACNA1C, PPP2R5D, PHF21A, DYNC1H1, UBR1, FOXP2, GABRB3, FOXP1, GABRB2, MKX, TRAF7, ANK2, KMT2C, NCOA1, CORO1A, PER1, ESR1, PPP5C, LDB1, NDEL1, CHD8, TBL1XR1, CACNA1E, STXBP1, CACNA2D3, CTNNB1, ARNTL, NRCAM, PHF12, MED13L, SCN2A, NPAS2, TSC22D4, ARID1B, TCF7L2, SCN1A, NUP54, CREBBP, HTR1B
Negative regulation of cellular macromolecule biosynthetic process	CTBP1, ESR1, SIN3A, SMARCC2, LDB1, PHF21A, CHD8, TBL1XR1, FOXP2, FOXP1, CTNNB1, ARNTL, PHF12, TSC22D4, TCF7L2, PER1, CREBBP
Negative regulation of macromolecule biosynthetic process	CTBP1, ESR1, SIN3A, SMARCC2, LDB1, PHF21A, CHD8, TBL1XR1, FOXP2, FOXP1, CTNNB1, ARNTL, PHF12, TSC22D4, TCF7L2, PER1, CREBBP
Negative regulation of cellular biosynthetic process	CTBP1, ESR1, SIN3A, SMARCC2, LDB1, PHF21A, CHD8, TBL1XR1, FOXP2, FOXP1, CTNNB1, ARNTL, PHF12, TSC22D4, TCF7L2, PER1, CREBBP
Transcription, DNA‐templated	CTBP1, PPP5C, TCF4, ESR1, SIN3A, SMARCC2, LDB1, PHF21A, CHD8, TBL1XR1, FOXP2, FOXP1, CTNNB1, MKX, ARNTL, PHF12, MED13L, NPAS2, TSC22D4, KMT2C, ARID1B, NCOA1, TCF7L2, PER1, CREBBP
Nucleic acid‐templated transcription	CTBP1, PPP5C, TCF4, ESR1, SIN3A, SMARCC2, LDB1, PHF21A, CHD8, TBL1XR1, FOXP2, FOXP1, CTNNB1, MKX, ARNTL, PHF12, MED13L, NPAS2, TSC22D4, KMT2C, ARID1B, NCOA1, TCF7L2, PER1, CREBBP
RNA biosynthetic process	CTBP1, PPP5C, TCF4, ESR1, SIN3A, SMARCC2, LDB1, PHF21A, CHD8, TBL1XR1, FOXP2, FOXP1, CTNNB1, MKX, ARNTL, PHF12, MED13L, NPAS2, TSC22D4, KMT2C, ARID1B, NCOA1, TCF7L2, PER1, CREBBP
Negative regulation of biosynthetic process	CTBP1, ESR1, SIN3A, SMARCC2, LDB1, PHF21A, CHD8, TBL1XR1, FOXP2, FOXP1, CTNNB1, ARNTL, PHF12, TSC22D4, TCF7L2, PER1, CREBBP
Regulation of transcription, DNA‐templated	CTBP1, TCF4, ESR1, SIN3A, SMARCC2, LDB1, PHF21A, CHD8, TBL1XR1, FOXP2, FOXP1, CTNNB1, MKX, ARNTL, PHF12, MED13L, NPAS2, TSC22D4, KMT2C, ARID1B, NCOA1, TCF7L2, PER1, CREBBP
Regulation of nucleic acid‐templated transcription	CTBP1, TCF4, ESR1, SIN3A, SMARCC2, LDB1, PHF21A, CHD8, TBL1XR1, FOXP2, FOXP1, CTNNB1, MKX, ARNTL, PHF12, MED13L, NPAS2, TSC22D4, KMT2C, ARID1B, NCOA1, TCF7L2, PER1, CREBBP
Regulation of transport	GFAP, CACNA1C, NDEL1, DYNC1H1, CACNA1E, STXBP1, CACNA2D3, CTNNB1, ARNTL, ANK2, SCN2A, CORO1A, TCF7L2, NUP54, PER1, SCN1A, HTR1B
Regulation of RNA biosynthetic process	CTBP1, TCF4, ESR1, SIN3A, SMARCC2, LDB1, PHF21A, CHD8, TBL1XR1, FOXP2, FOXP1, CTNNB1, MKX, ARNTL, PHF12, MED13L, NPAS2, TSC22D4, KMT2C, ARID1B, NCOA1, TCF7L2, PER1, CREBBP
Nervous system development	TCF4, SIN3A, SMARCC2, GFAP, LDB1, PPP2R5D, NDEL1, CHD8, STXBP1, FOXP2, GABRB3, GABRB2, CTNNB1, ARNTL, NRCAM, ANK2, NPAS2, SCN2A, ARID1B, NCOA1
Regulation of localization	SIN3A, GNAI1, GFAP, CACNA1C, LDB1, NDEL1, CACNA1E, DYNC1H1, STXBP1, FOXP1, CACNA2D3, CTNNB1, ARNTL, ANK2, SCN2A, CORO1A, TCF7L2, SCN1A, NUP54, PER1, HTR1B
Regulation of ion transport	CTNNB1, CACNA1C, ANK2, SCN2A, CACNA1E, STXBP1, CORO1A, SCN1A, PER1, CACNA2D3, HTR1B
Chromosome organization	CTBP1, ESR1, CTNNB1, SIN3A, SMARCC2, NUP155, LDB1, PHF21A, CHD8, TBL1XR1, KMT2C, ARID1B, TCF7L2
Cell–cell signaling	STX11, GFAP, CACNA1C, LDB1, CHD8, TBL1XR1, CACNA1E, STXBP1, GABRB3, CTNNB1, GABRB2, NUP155, ARNTL, ANK2, TCF7L2, HTR1B
Positive regulation of transcription by RNA polymerase II	TCF4, ESR1, CTNNB1, SIN3A, ARNTL, LDB1, CHD8, TBL1XR1, NPAS2, KMT2C, NCOA1, TCF7L2, PER1, CREBBP
Protein acetylation	CTBP1, SIN3A, ARNTL, NCOA1, LDB1, PER1, CREBBP
Positive regulation of transcription, DNA‐templated	TCF4, ESR1, SIN3A, SMARCC2, LDB1, CHD8, TBL1XR1, CTNNB1, ARNTL, NPAS2, KMT2C, ARID1B, NCOA1, TCF7L2, PER1, CREBBP
Positive regulation of nucleic acid‐templated transcription	TCF4, ESR1, SIN3A, SMARCC2, LDB1, CHD8, TBL1XR1, CTNNB1, ARNTL, NPAS2, KMT2C, ARID1B, NCOA1, TCF7L2, PER1, CREBBP
Positive regulation of RNA biosynthetic process	TCF4, ESR1, SIN3A, SMARCC2, LDB1, CHD8, TBL1XR1, CTNNB1, ARNTL, NPAS2, KMT2C, ARID1B, NCOA1, TCF7L2, PER1, CREBBP
Nucleobase‐containing compound biosynthetic process	CTBP1, PPP5C, TCF4, ESR1, SIN3A, SMARCC2, LDB1, PHF21A, CHD8, TBL1XR1, FOXP2, FOXP1, CTNNB1, MKX, ARNTL, PHF12, MED13L, NPAS2, TSC22D4, KMT2C, ARID1B, NCOA1, TCF7L2, PER1, CREBBP
Cellular response to organic cyclic compound	ESR1, GABRB2, CTNNB1, SIN3A, GNAI1, ARNTL, GABRB3, PER1, FOXP1, HTR1B
Action potential	SCN2A, CACNA1C, NRCAM, NUP155, SCN1A, ANK2
Response to organic cyclic compound	PPP5C, ESR1, GABRB2, CTNNB1, SIN3A, GNAI1, ARNTL, STXBP1, GABRB3, PER1, FOXP1, HTR1B
Regulation of RNA metabolic process	CTBP1, TCF4, ESR1, SIN3A, SMARCC2, LDB1, PHF21A, CHD8, TBL1XR1, FOXP2, FOXP1, CTNNB1, MKX, ARNTL, PHF12, MED13L, NPAS2, TSC22D4, KMT2C, ARID1B, NCOA1, TCF7L2, PER1, CREBBP
Heterocycle biosynthetic process	CTBP1, PPP5C, TCF4, ESR1, SIN3A, SMARCC2, LDB1, PHF21A, CHD8, TBL1XR1, FOXP2, FOXP1, CTNNB1, MKX, ARNTL, PHF12, MED13L, NPAS2, TSC22D4, KMT2C, ARID1B, NCOA1, TCF7L2, PER1, CREBBP
Positive regulation of cellular process	CTBP1, PPP5C, TCF4, ESR1, SIN3A, SMARCC2, GNAI1, GFAP, LDB1, PPP2R5D, NDEL1, CHD8, TBL1XR1, DYNC1H1, STXBP1, FOXP1, CTNNB1, TRAF7, NRCAM, ARNTL, ANK2, NPAS2, KMT2C, ARID1B, NCOA1, CORO1A, TCF7L2, PER1, CREBBP, HTR1B
Aromatic compound biosynthetic process	CTBP1, PPP5C, TCF4, ESR1, SIN3A, SMARCC2, LDB1, PHF21A, CHD8, TBL1XR1, FOXP2, FOXP1, CTNNB1, MKX, ARNTL, PHF12, MED13L, NPAS2, TSC22D4, KMT2C, ARID1B, NCOA1, TCF7L2, PER1, CREBBP
Membrane depolarization during action potential	SCN2A, CACNA1C, SCN1A, ANK2
Cellular response to endogenous stimulus	ESR1, GABRB2, CTNNB1, SIN3A, ARNTL, NDEL1, UBR1, NCOA1, CORO1A, GABRB3, PER1, FOXP1, CREBBP, HTR1B
Regulation of cellular localization	CTNNB1, SIN3A, GNAI1, CACNA1C, NDEL1, ANK2, DYNC1H1, STXBP1, CORO1A, TCF7L2, NUP54
Positive regulation of RNA metabolic process	TCF4, ESR1, SIN3A, SMARCC2, LDB1, CHD8, TBL1XR1, CTNNB1, ARNTL, NPAS2, KMT2C, ARID1B, NCOA1, TCF7L2, PER1, CREBBP
Multicellular organismal signaling	SCN2A, CACNA1C, NRCAM, NUP155, SCN1A, ANK2
Protein acylation	CTBP1, SIN3A, ARNTL, NCOA1, LDB1, PER1, CREBBP
Response to endogenous stimulus	ESR1, GABRB2, CTNNB1, SIN3A, GNAI1, ARNTL, NDEL1, UBR1, NCOA1, CORO1A, GABRB3, PER1, FOXP1, CREBBP, HTR1B
Histone modification	CTBP1, CTNNB1, SIN3A, LDB1, TBL1XR1, KMT2C, NCOA1, PER1, CREBBP
Organic cyclic compound biosynthetic process	CTBP1, PPP5C, TCF4, ESR1, SIN3A, SMARCC2, LDB1, PHF21A, CHD8, TBL1XR1, FOXP2, FOXP1, CTNNB1, MKX, ARNTL, PHF12, MED13L, NPAS2, TSC22D4, KMT2C, ARID1B, NCOA1, TCF7L2, PER1, CREBBP
Histone acetylation	CTBP1, SIN3A, NCOA1, LDB1, PER1, CREBBP
Regulation of cellular macromolecule biosynthetic process	CTBP1, TCF4, ESR1, SIN3A, SMARCC2, LDB1, PHF21A, CHD8, TBL1XR1, FOXP2, FOXP1, CTNNB1, MKX, ARNTL, PHF12, MED13L, NPAS2, TSC22D4, KMT2C, ARID1B, NCOA1, TCF7L2, PER1, CREBBP
Internal peptidyl‐lysine acetylation	CTBP1, SIN3A, NCOA1, LDB1, PER1, CREBBP
Regulation of macromolecule biosynthetic process	CTBP1, TCF4, ESR1, SIN3A, SMARCC2, LDB1, PHF21A, CHD8, TBL1XR1, FOXP2, FOXP1, CTNNB1, MKX, ARNTL, PHF12, MED13L, NPAS2, TSC22D4, KMT2C, ARID1B, NCOA1, TCF7L2, PER1, CREBBP
Internal protein amino acid acetylation	CTBP1, SIN3A, NCOA1, LDB1, PER1, CREBBP
Regulation of nucleobase‐containing compound metabolic process	CTBP1, TCF4, ESR1, SIN3A, SMARCC2, LDB1, PHF21A, CHD8, TBL1XR1, FOXP2, FOXP1, CTNNB1, MKX, ARNTL, PHF12, MED13L, NPAS2, TSC22D4, KMT2C, ARID1B, NCOA1, TCF7L2, PER1, CREBBP
Peptidyl‐lysine modification	CTBP1, CTNNB1, SIN3A, LDB1, KMT2C, NCOA1, PER1, CREBBP
Positive regulation of macromolecule biosynthetic process	TCF4, ESR1, SIN3A, SMARCC2, LDB1, CHD8, TBL1XR1, CTNNB1, ARNTL, NPAS2, KMT2C, ARID1B, NCOA1, TCF7L2, PER1, CREBBP
Protein localization to nuclear inner membrane	NUP155, NUP54
Canonical Wnt signaling pathway involved in positive regulation of epithelial to mesenchymal transition	CTNNB1, TCF7L2
Peptidyl‐lysine acetylation	CTBP1, SIN3A, NCOA1, LDB1, PER1, CREBBP
Multicellular organismal process	TCF4, SIN3A, SMARCC2, GFAP, CACNA1C, PPP2R5D, DYNC1H1, FOXP2, GABRB3, FOXP1, GABRB2, MKX, NUP155, ANK2, NCOA1, CORO1A, PER1, ESR1, LDB1, CHD8, NDEL1, TBL1XR1, STXBP1, CTNNB1, ARNTL, NRCAM, C3orf62, NPAS2, SCN2A, ARID1B, TCF7L2, SCN1A, CREBBP, HTR1B
Cardiac muscle cell action potential involved in contraction	CACNA1C, NUP155, SCN1A, ANK2
Chromatin organization	CTBP1, ESR1, SIN3A, SMARCC2, PHF21A, CHD8, TBL1XR1, KMT2C, ARID1B
Regulation of membrane potential	GABRB2, CACNA1C, NRCAM, NUP155, ANK2, SCN2A, GABRB3, SCN1A
System development	TCF4, ESR1, SIN3A, SMARCC2, GFAP, CACNA1C, LDB1, PPP2R5D, NDEL1, CHD8, TBL1XR1, STXBP1, FOXP2, GABRB3, FOXP1, GABRB2, CTNNB1, MKX, ARNTL, NRCAM, ANK2, SCN2A, NPAS2, ARID1B, NCOA1, TCF7L2
Regulation of cellular biosynthetic process	CTBP1, TCF4, ESR1, SIN3A, SMARCC2, LDB1, PHF21A, CHD8, TBL1XR1, FOXP2, FOXP1, CTNNB1, MKX, ARNTL, PHF12, MED13L, NPAS2, TSC22D4, KMT2C, ARID1B, NCOA1, TCF7L2, PER1, CREBBP
Positive regulation of nucleobase‐containing compound metabolic process	TCF4, ESR1, SIN3A, SMARCC2, LDB1, CHD8, TBL1XR1, CTNNB1, ARNTL, NPAS2, KMT2C, ARID1B, NCOA1, TCF7L2, PER1, CREBBP
Multicellular organism development	TCF4, ESR1, SIN3A, SMARCC2, GFAP, CACNA1C, LDB1, PPP2R5D, NDEL1, CHD8, TBL1XR1, STXBP1, FOXP2, GABRB3, FOXP1, GABRB2, CTNNB1, MKX, ARNTL, NRCAM, ANK2, SCN2A, NPAS2, ARID1B, NCOA1, TCF7L2, CREBBP

### 3.5 Network decomposition analysis (NDA)

Network decomposition analysis (NDA) involves breaking down a network into fundamental pathways that capture the time‐invariant topological structure. NDA serves the purpose of defining the overall capabilities of a cell. To conduct a detailed analysis of information flow, the CPPIN is dissected into linear pathways connecting transcription factors and various proteins within the PPI network. Linear pathways originating from the central circadian gene product (ARNTL [BMAL1]) to the transcriptional factor gene (TCF4) are examined using the NetSearch Algorithm. TCF4 functions as a transcription factor that regulates a wide array of developmental processes. Deficiencies in TCF4 have been linked to various human disorders, including Pitt–Hopkins syndrome, an ID condition, and ASD. The total count of pathways from ARNTL to TCF4 is estimated to be 1350.

**FIGURE 4:**
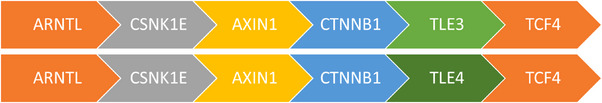
Six‐protein length pathways of combined circadian–autism protein–protein interaction network (CAPPIN) from ARNTL to TCF4 (CPPIN: ARNTL and CSNK1E (hub/bottleneck proteins), AXIN1, CTNNB1, TLE3, TLE4; APPIN: CTNNB1, TCF4). This figure illustrates the 6‐steps pathways within the CAPPIN that connect ARNTL to TCF4. The central role of ARNTL and CSNK1E as hub/bottleneck proteins is highlighted, along with the significant involvement of AXIN1, CTNNB1, TLE3, and TLE4 in these pathways.

**FIGURE 5:**
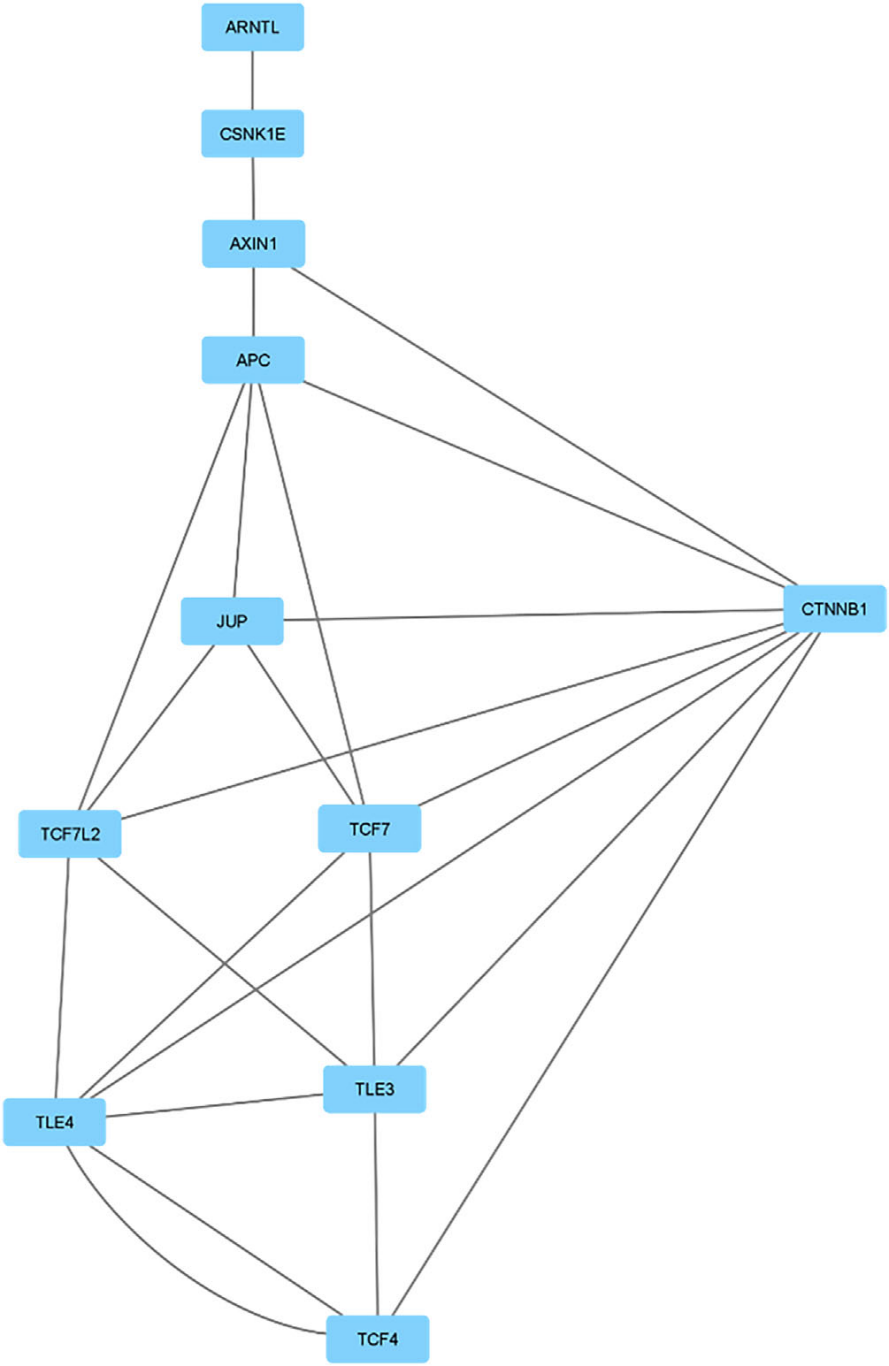
The most likely pathways of combined circadian–autism protein–protein interaction network (CAPPIN) (5–6–7–8 steps) from ARNTL to TCF4. This figure illustrates the most likely pathways within the CAPPIN, depicting a series of 5–6–7–8 steps that connect ARNTL to TCF4. CAPPIN represents a combined network involving critical proteins such as ARNTL, CSNK1E (hub/bottleneck proteins), and TCF4. These pathways provide valuable insights into the complex protein–protein interactions that underlie the connection between circadian rhythms and autism spectrum disorder (ASD).

The participation of proteins in these linear paths indicates their significance in signal transduction, as any signaling network is composed of linear paths. Therefore, the participation percentages of each protein in linear paths of the CAPPIN are calculated to gain insights into the roles of these proteins in transmitting information flow from ARNTL to TCF4. The proteins most frequently encountered in these linear paths are identified by MATLAB. Among these outcomes, CSNK1E protein is recognized as the most interactive protein with the initial ARNTL protein. Extending this procedure to all proteins with path lengths of 6, 7, and 8, the AXIN1 protein emerges as the most interactive protein with CSNK1E. The characteristic path length of CAPPIN was determined to be 5.896. Therefore, the pathways of 6 steps are visualized in Figure 4, whereas the most probable outcomes of pathways with steps of 5–6–7–8 (path lengths) are depicted in Figure 5.

Bottleneck proteins, recognized as central connectors that are crucial to numerous shortest paths in an interaction network, control most information flow (signal transduction) from ARNTL to the TCF4 protein. ARNTL and CSNK1E function as the hub/bottleneck proteins in the network. Thus, mutations in these proteins may potentially disrupt the circadian system leading to disorders, including ASD. A recent study on AXIN1 indicates a correlation with autistic traits (Smedler et al., 2021). The role of CTNNB1 gene in the regulation of cognitive and autistic‐like behaviors has been reported in several publications (Dong et al., 2016). TLE3 is a target of FOXP2, a transcription factor located on chromosome 7q31 related to autistic disorder (Benítez‐Burraco et al., 2018).

## 4 DISCUSSION

### 4.1 Circadian clock crosstalks with many pathways

The human body possesses a circadian rhythm that regulates numerous physiological activities across various organizational levels, maintaining a 24‐h transcription‐translation feedback loop. There are nine core gene products associated with circadian rhythms: Period 1 (PER1), Period 2 (PER2), Period 3 (PER3), Casein Kinase Iε, CLOCK, ARNTL (BMAL1), TIM, CRY1, and CRY2 (Lévi, 2002). These gene products also regulate cell proliferation and could play a critical role in the cell cycle. Furthermore, studies on micro‐peripherical tissues suggest that the biological clock is activated in response to DNA damage. Particularly, PER2 is implicated in the DNA‐damage response process in rodents (Fu & Lee, 2003).

In the regulation of circadian rhythms, the BMAL1 (ARNTL) gene that interacts with the CLOCK or NPAS2 genes gives rise to the formation of BMAL1:NPAS2 or BMAL1:CLOCK complexes within the cytoplasm. These resultant complexes play a role in activating various other circadian genes within the nucleus, working in conjunction with the E‐box: PER1/2, CRY1/2, and DEC1/2. PER1/2‐CRY1/2 and DEC1/2 complexes function to inhibit the activity of heterodimeric transcription factors BMAL1:NPAS2 or BMAL1:CLOCK, and this inhibition varies based on the tissue. Consequently, these heterodimers participate in initiating their transcription in a negative loop (Li, 2019).

**FIGURE 6:**
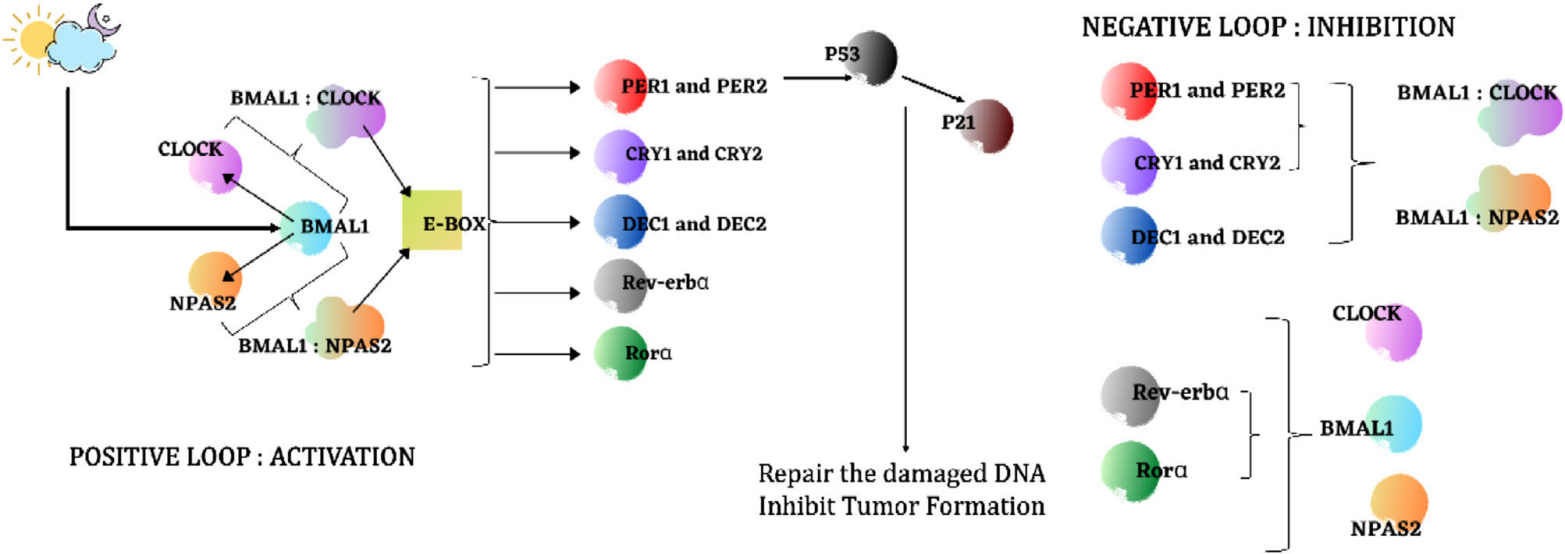
Mammalian protein signaling pathway with positive and negative feedback loops. This figure illustrates circadian genes and their feedback loops in the regulation of mammalian circadian rhythms, with positive and negative feedback mechanisms. It also highlights the pivotal role of the P53 tumor suppressor gene.

An important point to note is the influence of the P53 gene by PER1 and PER2. Specifically, PER2 directly interacts with P53, preventing its ubiquitination and thereby leading to P53 stabilization and modulation of its nuclear import (Gotoh et al., 2015, 2014, 2016). Notably, the absence of P53 can result in altered PER2 expression within the SCN (Miki et al., 2013). Both P53 and its downstream effector P21 (activated by P53) play pivotal roles in DNA damage repair pathways and the inhibition of tumor formation (Li, 2019). Examining the main circadian pathway (as depicted in Figure 6), it is evident that impaired circadian genes or gene products fail to activate P53, which, in reality, functions to suppress tumor formation.

BMAL1:CLOCK heterodimer whose activation is modulated by CREBBP functions as a transcriptional activator within the circadian rhythm and plays a pivotal role in regulating the expression of circadian genes (Lee et al., 2010). However, the MYC protein, recognized as an oncogene, disrupts the formation of the BMAL1‐CLOCK dimers by binding to the E‐box. Furthermore, MYC takes part in activating another negative regulator REV‐ERB, leading to suppressing BMAL1 expression and consequently impacting the circadian clock (Altman et al., 2015). A well‐functioning circadian rhythm is indispensable for maintaining good health.

### 4.2 Dysfunction of metabolic pathways due to mutations in the clock‐controlled genes

Autism is influenced by the dysfunction of significant pathways within the CNS. NPAS2, also known as a member of PAS protein 4, is a transcription factor protein that plays a crucial role in sleep homeostasis and maintaining circadian behaviors under normal light/dark conditions. NPAS2 protein emerges prominently in clusters of excessively associated proteins (MCODE clusters), as well as in the result of GO enrichment analysis. Impaired memory and sleep disturbances (symptomatic of ASD) have been investigated using mouse models. Npas2 (−/−) knockout mice have shown disruptions in complex emotional memory (Garcia et al., 2000), indicating the critical role of non‐REM sleep homeostasis and a reduction in total sleep time (Franken et al., 2006). Furthermore, a specific protein variant, NPAS2 471 Leu/Ser, has been associated with seasonal affective disorder and diurnal preference in humans (Johansson et al., 2003). In the case of impaired transcription/translation feedback loop, the binding of CLOCK:BMAL1 or NPAS2:BMAL1 is inhibited, resulting in the downregulation of both the period and cryptochrome genes’ transcription (Lorsung et al., 2021; Ye et al., 2014).

Mutations in the clock‐controlled genes are frequently observed in individuals with ASD. Although individuals with ASD have been studied for genes strongly associated with circadian rhythm, statistical analyses have revealed variations among these ASD individuals (Yang et al., 2016). The polymorphisms in Npas2 such as cytosine/thymine SNP in intron 3 (NPAS2_X3_C_T) (Nicholas et al., 2007), mutations in PER1 including cytosine → guanine SNP (Per1_rs885747) and a cytosine/adenine SNP (Per1_rs6416892) (Nicholas et al., 2007), as well as mutations in PER2 (proline/alanine substitution at amino acid 1228), and mutations in PER3 (an arginine/glutamine substitution at amino acid 366) have been identified in individuals with ASD. These variations have a detrimental impact on gene function. These clinical findings underscore the association of circadian rhythm genes/gene products PER2 and PER3 in the pathogenesis of ASD. Additionally, the mutations in NR1D1 have also been linked to abnormal ASD brain development (Lorsung et al., 2021). RORα/β is known to activate the transcription of BMAL1 and NFIL3 (Preitner et al., 2002; Ueda et al., 2005). However, RORα cannot function properly in the case of impaired NR1D1. Another pathway linked to ASD is the canonical WNT/β‐catenin pathway which becomes upregulated under the dysregulation of circadian rhythm (sleep disorder) (Vallée et al., 2020). This pathway initiates the metabolic reprogramming of cellular energy metabolism in individuals with developmental cognitive disorders (Vallée et al., 2020).

#### 4.2.1 Oxidative stress and its link to ASD and circadian clock

Oxidative stress is a mechanism through which prenatal and postnatal problems contribute to the development of ASD. In more than 100 publications investigating any relationship between oxidative stress and ASD/Rett syndrome, an association has been consistently reported, and these studies have explored abnormalities in microglial activation, maternal antibodies to fetal brain tissue, genetic mutations affecting the immune system, and cytokine abnormalities, all linking oxidative stress with ASD. The effects of oxidative stress on ASD provide insights into the influence of parental age on ASD. Older men's spermatozoa are more vulnerable to oxidative stress, potentially leading to DNA fragmentation, a factor associated with neuropsychiatric disorders (Mandic‐Maravic et al., 2019).

Due to a weakened antioxidant defense mechanism, children with ASD seem to be more susceptible to oxidative stress, leading to elevated lipid peroxidation. This observation suggests that early antioxidant treatment could potentially enhance prognosis by mitigating oxidative stress before it inflicts further irreversible brain damage (Bjørklund et al., 2020).

From blood pressure and sleep/wake cycles to cellular signaling pathways that play crucial roles in health and diseases, oxygen and circadian rhythmicity are essential for maintaining homeostasis in various physiological processes. The ability of the human body or cells to regulate internal systems, such as redox levels and circadian rhythms, could be compromised when subjected to significant stress. Impairment in redox regulation and circadian rhythms can lead to various adverse consequences at both the cellular and organismal levels, including the development of heart disease, neurodegenerative conditions, and cancer (Fanjul‐Moles &López‐Riquelme, 2016; Lananna & Musiek, 2020; Wilking et al., 2013). The impacts of oxidative stress and dysregulated circadian rhythms have been extensively studied, and the molecular mechanisms that connect these two processes are well established and observed in the reconstructed networks of the present research.

### 4.3 The connection between the circadian clock and autism within mTor signaling pathway

The mTOR signaling pathway plays an important role in controlling significant functions within neurons, encompassing neurodevelopment and intracellular metabolism (Liu & Sabatini, 2020; Wullschleger et al., 2006). Recent studies (Cao, 2018; Cao et al., 2011) have demonstrated the influence of the circadian clock on mTOR activities. These activities exhibit robust diurnal oscillations across various systems. Furthermore, mTOR signaling impacts the duration, synchronization, and entrainment of central and peripheral circadian clocks (Singla, Mishra, Cao, 2022; Singla, Mishra, Lin, et al. 2022). The study, which focused on the association between autism and circadian genes in mice, demonstrated that mice lacking the Bmal1 gene exhibited differences in the expression of genes associated with autism and ataxia and also displayed dysregulated pathways, including overactive mTORC1 signaling (Liu et al., 2022). Moreover, mTOR creates a complex signaling network that combines external and internal signals to influence mRNA translation (Liu & Sabatini, 2020; Wullschleger et al., 2006).

The study on autism (Neves‐Pereira et al., 2009) demonstrates that the eukaryotic initiation factor 4E (EIF4E) gene, which plays a role in translation control, is implicated in the disorder. Specifically, specific mutations in the EIF4E promoter region have been linked to autism. In the context of APPIN5, EIF4E serves as a hub protein within the autism network, while also being closely interconnected with the mTOR signaling pathway and the circadian clock. One of the downstream processes of this pathway is the activation of the EIF4E, a protein responsible for binding to the mRNA cap structure (Romagnoli et al., 2021). The results of this study point to a need on a thorough evaluation of the mTOR pathway and a review of ribosomal proteins.

### 4.4 Ribosome biogenesis and its relation to ASD and circadian clock

The GO enrichment analyses highlight the significance of ribosome biogenesis in relation to ASD that could potentially contribute to its causative factors and related neurodevelopmental disorders. In autism, the expression of ribosomal protein‐encoding genes is notably elevated (upregulated), suggesting an intensified ribosome biogenesis process and consequently heightened protein synthesis (Lombardo, 2021). The increased copy number of ribosomal genes within the dendrites alters the level of dendritic translation, thereby representing a risk factor for neuropsychiatric disorders (Porokhovnik & Lyapunova, 2019). As a result of disturbed “translational homeostasis,” individuals with abundant ribosomes (due to moderate or high copy numbers of ribosomal genes) may develop ASD. In a study by Tran et al. (2020), new variants have been identified in six candidate genes (CHM, ENPP1, IGF1, LAS1L, SYP, and TBX22) enriched in an ASD cohort. These genes are associated with various neurological conditions and play roles in neuronal biological processes, such as synaptic processes, regulation of transport, and ribosome maturation. Therefore, the genetic predispositions within these genes are reported to be causative factors for ASD (Tran et al., 2020). Another ribosomal gene with missense mutations is RPL10 gene, which exhibits high expression in mouse hippocampus—a critical site of action for learning, memory, and social and affective functions. Consequently, functionally altered RPL10 protein may contribute to cognitive malfunction, which is also observed in autism (Klauck, 2006). On the other hand, another study suggests that RPL10 might not have a significant impact on ASD susceptibility (Gong et al., 2009).

The circadian clock regulates the transcription of translation initiation factors and orchestrates the clock‐dependent rhythmic activation of signaling pathways involved in their regulation. This intricate process governs the temporal translation of a subset of mRNAs essential for ribosome biogenesis. Consequently, the circadian clock plays a pivotal role in synchronizing the transcription and translation processes crucial for ribosome biogenesis (Benegiamo et al., 2016; Jouffe et al., 2013). Recent research has examined the downregulated genes for RNA‐binding proteins in the transcriptome of induced pluripotent stem cell neurons (iNeurons) to model the RTS (Larizza et al., 2022). Intriguingly, many individuals with RTS display traits similar to those observed in individuals with autism. This study has identified several downregulated gene products in RTS, including ribosomal subunits and nucleolar proteins such as NOP58 and fibrillarin. These proteins form complexes with snoRNAs and play a key role in driving posttranscriptional changes required for rRNA maturation. Fibrillarin is essential for the epigenetic regulation of ribosomal genes, and NOP58‐associated snoRNA levels are modulated by NOP58 interactor BMAL1, a transcriptional regulator of the circadian rhythm.

Despite these findings, the precise involvement of ribonucleoprotein particles in the production of rhythmic gene expression remains a mystery. Informatics aimed at integrating these findings with circadian transcriptome datasets are required to elucidate the posttranscriptional processes underlying circadian control, whereas the target RNAs for many of the RNA‐binding proteins are being discovered.

## 5 CONCLUSION

Autism is a complex disorder involving over 1000 genes. Alongside genetic factors, various environmental influences contributing to ASD have been identified, including maternal nutritional factors and obesity, maternal stress, maternal immunological response/dysfunction, early‐life immune insults, parental age, heavy metals, and air pollution. In the present study, a network‐based approach is conducted to identify significant pathways and genes/gene products within both individuals (pure autistic and pure circadian) and combined networks of circadian rhythm and ASD. Numerous key hub gene products are highlighted, showing significant enrichment in areas such as ribosomal proteins, protein synthesis, regulatory pathways, and the Wnt/β‐catenin signaling pathway. These findings merit further investigation through experimental validation studies. Several fundamental processes that pertain to synaptic function are also revealed to be regulated by specific genes associated with ASD. These processes encompass ribosome maturation and mRNA regulation. Thus, changes in synaptic function and gene regulation may serve as causative factors for ASD. Given that the translation of significant proteins associated with ASD occurs within the synaptic region, maintaining translational homeostasis could potentially prevent or mitigate this complex and multifactorial disorder.

The enriched GO terms for combined network CAPPIN are primarily related to cytoplasmic translation or translation, glucocorticoid receptor signaling pathway and its regulation, ribonucleoprotein complex biogenesis, cellular response to endogenous stimulus, and processes such as histone acetylation and peptidyl‐lysine acetylation. Histone deacetylases have recently been implicated in ASD and other neurodevelopmental disorders characterized by similar clinical and diagnostic comorbidities (e.g., epilepsy, anxiety, and intellectual impairment). The application of epigenetic drugs, such as histone deacetylase inhibitors (Larizza et al., 2022) in the treatment of neurodevelopmental disorders arising from RNA‐binding proteins could potentially restore the activity of lysine (K) acetyltransferases (KAT) in various neurodegenerative conditions, including autism.

The present study introduces a novel perspective on metabolic mechanisms and unveils potential key proteins/pathways such as ribosome biogenesis, redox, and oxidative stress. Consequently, it highlights that it could mitigate the development of ASD triggered by environmental factors like maternal stress, or advanced parental age (which is linked to spermatozoids of older men and oxidative stress). Ultimately, comprehending the connection between circadian rhythm and ASD provides valuable insights into the role of these pathways in the etiology and pathogenesis of ASD. Initiating antioxidant treatment early on could potentially enhance the trajectory of the medical condition by mitigating oxidative stress before it leads to irreversible brain damage. Furthermore, emerging treatments are under investigation, targeting epigenetic marks like DNA methylation and histone acetylation, with the hope of addressing or preventing autism.

### PEER REVIEW

The peer review history for this article is available at https://publons.com/publon/10.1002/brb3.3273.

## Data Availability

Data openly available in a public repository that issues datasets with DOIs: 10.1016/j.cell.2019.07.037; 10.1093/nar/gkj109; 10.1093/bioinformatics/btn615; 10.1093/nar/gkw1028; 10.1038/ncomms4650.
